# Alterations of lipid‐mediated mitophagy result in aging‐dependent sensorimotor defects

**DOI:** 10.1111/acel.13954

**Published:** 2023-08-23

**Authors:** Natalia Oleinik, Onder Albayram, Mohamed Faisal Kassir, F. Cansu Atilgan, Chase Walton, Eda Karakaya, John Kurtz, Alexander Alekseyenko, Habeeb Alsudani, Megan Sheridan, Zdzislaw M. Szulc, Besim Ogretmen

**Affiliations:** ^1^ Department of Biochemistry and Molecular Biology Medical University of South Carolina Charleston South Carolina USA; ^2^ Hollings Cancer Center Medical University of South Carolina Charleston South Carolina USA; ^3^ Departments of Pathology and Laboratory Medicine Medical University of South Carolina Charleston South Carolina USA; ^4^ Department of Neuroscience Medical University of South Carolina Charleston South Carolina USA; ^5^ Public Health Medical University of South Carolina Charleston South Carolina USA; ^6^ Cancer Center Cold Spring Harbor Laboratory Cold Spring Harbor New York USA

**Keywords:** aging, ceramide, CerS1, Drp1, mitochondrial metabolism, mitophagy, neurodegeneration, sensorimotor defects

## Abstract

The metabolic consequences of mitophagy alterations due to age‐related stress in healthy aging brains versus neurodegeneration remain unknown. Here, we demonstrate that ceramide synthase 1 (CerS1) is transported to the outer mitochondrial membrane by the p17/PERMIT transporter that recognizes mislocalized mitochondrial ribosomes (mitoribosomes) via 39‐FLRN‐42 residues, inducing ceramide‐mediated mitophagy. P17/PERMIT‐CerS1‐mediated mitophagy attenuated the argininosuccinate/fumarate/malate axis and induced d‐glucose and fructose accumulation in neurons in culture and brain tissues (primarily in the cerebellum) of wild‐type mice in vivo. These metabolic changes in response to sodium‐selenite were nullified in the cerebellum of CerS1to/to (catalytically inactive for C18‐ceramide production CerS1 mutant), PARKIN−/− or p17/PERMIT−/− mice that have dysfunctional mitophagy. Whereas sodium selenite induced mitophagy in the cerebellum and improved motor‐neuron deficits in aged wild‐type mice, exogenous fumarate or malate prevented mitophagy. Attenuating ceramide‐mediated mitophagy enhanced damaged mitochondria accumulation and age‐dependent sensorimotor abnormalities in p17/PERMIT−/− mice. Reinstituting mitophagy using a ceramide analog drug with selenium conjugate, LCL768, restored mitophagy and reduced malate/fumarate metabolism, improving sensorimotor deficits in old p17/PERMIT−/− mice. Thus, these data describe the metabolic consequences of alterations to p17/PERMIT/ceramide‐mediated mitophagy associated with the loss of mitochondrial quality control in neurons and provide therapeutic options to overcome age‐dependent sensorimotor deficits and related disorders like amyotrophic lateral sclerosis (ALS).

## Graphical abstract

During healthy aging, reactive oxygen/nitrogen species (ROS/RNS) induce Drp1‐dependent mitochondrial fission, and ceramide synthase 1 (CerS1) transport to the outer mitochondrial membrane (OMM) by p17/PERMIT that recognizes mislocalized mitochondrial ribosomes through ICT1 inducing ceramide‐mediated mitophagy, clearing damaged mitochondria and decreasing malate/fumarate accumulation in neurons. Loss of p17/PERMIT enhances damaged mitochondria accumulation with increased malate/fumarate metabolism and age‐dependent sensorimotor abnormalities. Reinstituting mitophagy using a ceramide analog drug with selenium conjugate, LCL768 restores mitophagy and reduces malate/fumarate metabolism, improving sensorimotor deficits.

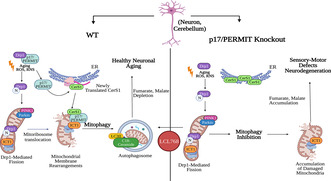

**Abbreviations**
A: adult mice (15–25 months old)
ACO2: aconitase 2
AD: Alzheimer's disease
ALS: amyotrophic lateral sclerosis
ASL: argininosuccinate lyase
ASNS: asparagine synthetase
ATP: adenosine triphosphate
Bcl‐2: B‐cell lymphoma 2
Bcl‐Xl: BCL2‐like protein
CCCP: m‐chlorophenyl hydrazone
CerS1: ceramide synthase 1
CerS1‐6: ceramide synthases 1‐6
CerS1to/to: functionally inactive CerS1 mutant
Cox‐IV: cytochrome c oxidase subunit IV
Cox‐17: cytochrome c oxidase copper chaperone COX17
CS: citrate synthase
Cys: cysteine
Cys‐NO: nitrosylated cysteine
DAPI: 4′,6‐diamidino‐2‐phenylindole
Drp1: Dynamin‐related protein 1
ECAR: extracellular acidification rate
EM: electron microphotograph
ER: endoplasmic reticulum
ER‐SURF: mitochondrial trafficking of proteins through the ER surface
FA: fumarate
FL: full length
H&E: hematoxylin, and eosin staining
IACUC: Institutional Animal Care and Use Committee
ICT1: immature colon carcinoma transcript
IMM: inner mitochondrial membrane
IMS: intermembrane space
IP: immunoprecipitation
LC3: microtubule‐associated protein
LS‐MS/MS: liquid chromatography with tandem mass spectrometry
M: mitochondrial fraction
MA: malate
MAP‐4: microtubule associated protein 4
mitoribosomes: mitochondrial ribosomes
MTG: mitotracker green
MTR: mitotracker red
NM: non‐mitochondrial fraction
NUBPL: nucleotide‐binding protein‐like
OCR: oxidative consumption
OMM: outer mitochondrial membrane
PD: Parkinson's disease
PK: proteinase K
p17/PERMIT: protein 17/protein endoplasmic reticulum mitochondrial transporter
p17KO: p17−/−, p17 knock‐out
PRKN: PARKIN
PRKNKO: PRKN−/−, PARKIN knock‐out
RB: ribosomal binding
Rbmut: ribosomal mutant
Rc: Pearson correlation coefficient (coefficient of colocolization)
Scr RNA: scrambled RNA
SD: standard deviation
SDHA: succinate dehydrogenase
SEM: standard error of the mean
shRNA: short hairpin RNA
siRNA: small interfering RNA
SoSe: Sodium Selenite
TEM: transmission electron microscope
TIM23: translocase of inner mitochondrial membrane 23
TMRE: Tetramethylrhodamine, ethyl ester
TOM20: translocase of outer mitochondrial membrane 20
TOM40: translocase of outer mitochondrial membrane 40
VDAC: Voltage dependent anion channel
WL: whole lysate
WT: wild‐type mice
W/V: weight per volume
Y: young mice (2.5‐month‐old)
3M: three months old
15M: fifteen months old

## 1 INTRODUCTION

Mitochondrial morphology and function are regulated by dynamic cellular processes, involving protein trafficking and import. Recruiting Drp1 (dynamin‐related protein) to the outer mitochondrial membrane (OMM) is critical to induce mitochondrial fission, resulting in rearrangements of mitochondrial membranes (Kleele et al., 2021; Kraus et al., 2021). Mitochondrial DNA encodes several genes translated by specialized ribosomes for proteins to be localized specifically to the inner mitochondrial membranes (IMM). The OMM contains proteins that regulate mitochondrial import and export of macromolecules, such as proteins and lipids. Bioactive sphingolipid ceramide synthesis is induced in response to various stress stimuli, including aging (Blom et al., 2015; Dadsena et al., 2019). Endogenous ceramides contain different fatty acyl chains generated by ceramide synthases 1‐6 (CerS1‐6) (Dany et al., 2016; Kim et al., 2022), such as CerS1‐generated C18‐ceramide. CerS1/C18‐ceramide‐dependent mitophagy (Smirnova et al., 2001) requires LC3 (microtubule‐associated light chain protein 3) and Drp1 (Ogretmen, 2018; Thomas et al., 2017). Recently, we discovered that in response to stress signaling mediated by sodium selenite, SoSe, a known inducer of mitophagy (Sentelle et al., 2012), newly synthesized CerS1 enzyme, rather than C18‐ceramide, is transported from the ER to damaged mitochondria by p17/PERMIT possibly through the ER‐SURF‐dependent (Hansen et al., 2018) transport to induce ceramide generation and mitophagy (Oleinik et al., 2019).

Mitochondria provide metabolic adaptation to high energy demands via tricarboxylic acid and oxidative phosphorylation by generating ATP and other macromolecules (Vasan et al., 2020). Impaired mitophagy leads to the accumulation of damaged mitochondria, which is associated with neurodegenerative disorders, such as Alzheimer's disease (AD) (Fang et al., 2019; Xie et al., 2022), Parkinson's disease (PD) (Vos et al., 2021; Youle & Narendra, 2011), or amyotrophic lateral sclerosis, ALS (Harding et al., 2021). Thus, restoring mitophagy provides a mechanism of mitochondrial quality control in neurons by removing damaged mitochondria during healthy brain aging. However, metabolic consequences of stress‐induced mitophagy in healthy neuronal aging versus neurodegeneration remain largely unknown.

Here, we set out experiments to understand aging‐dependent neurodegenerative and metabolic consequences of p17/PERMIT‐CerS1/C18‐ceramide‐mediated mitophagy alterations in multiple cell types and genetic mouse models. Our data provide new mechanism‐based therapeutic options to attenuate age‐dependent sensorimotor deficits in neurodegenerative disorders like ALS.

## 2 RESULTS

### 2.1 The p17/PERMIT‐CerS1 complex is recruited to the OMM by mislocalized mitochondrial ribosomes (mitoribosomes)

**FIGURE 1:**
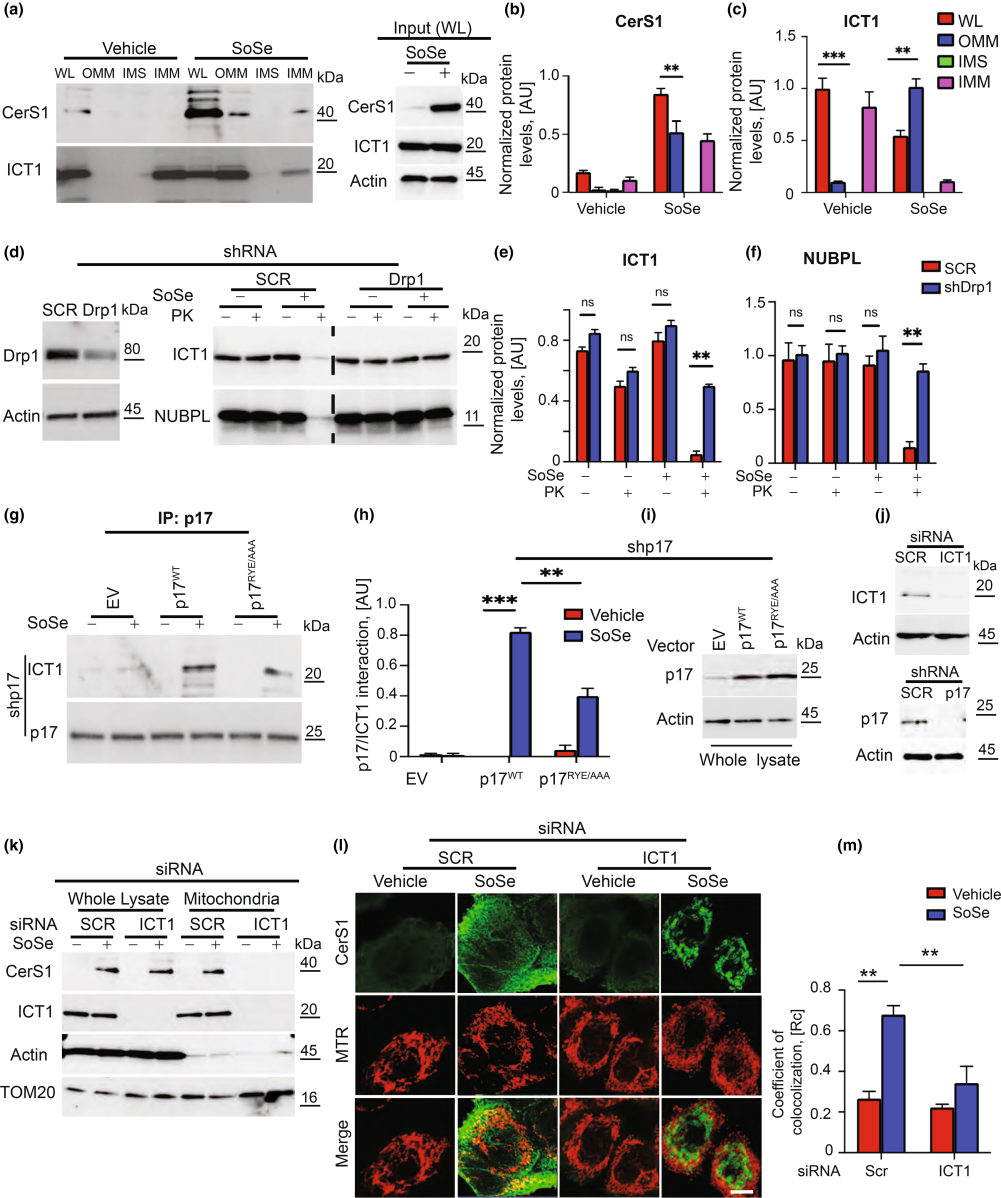
Mitochondrial membrane rearrangements are critical in recruiting p17/PERMIT‐CerS1 to mitochondria. (a) CerS1, and ICT1 in the whole lysate (WL) and mitochondrial fractions in vehicle (left) or SoSe (right) treated UM‐SCC‐1A cells. (b and c) Quantification of (a). Data are means ± SD (*n* = 3 independent experiments, **p < 0.01; ***p < 0.001). Normalization was done using corresponding protein levels. (d) Drp1 in cells treated with SCR control and Drp1 shRNA (left). Levels of ICT1 (top) and NUBPL (bottom) proteins in mitochondria isolated from SCR control and shDrp1 UM‐SCC‐1A cells treated with vehicle (−) or SoSe (+), 10 µM 3 h (right panel). (e and f) Quantification of (d). Data are means ± SD (*n* = 3 independent experiments, nsp > 0.5; **p < 0.01). (g) Representative Western Blot of Co‐IP analysis of ICT1 and p17 interaction in UM‐SCC‐1A cells with silenced endogenous p17 ectopically expressing empty vector (EV), wild type of p17 (WT) and p17RYE/AAA. (h) Quantification of p17/ICT1 interaction in cells from (g). Data are means ± SD (*n* = 3 independent experiments, ***p < 0.001). (i) Levels of p17WT and P17RYE/AAA mutant transiently expressed in p17 shRNA UM‐SCC‐1A cells. (j) ICT1 in UM‐SCC‐1A cells stably transfected with scrambled (Scr) or ICT1 shRNAs. (k) CerS1 and ICT1 in the whole lysates and mitochondrial fractions of Scr control and siICT1 RNA treated cells. (l) Confocal images of cells treated for 3 h with 10 μM of SoSe and stained for CerS1 (green) and MTR (red), mitochondrial marker. Yellow shows co‐localization. (m) Quantification of (l). Rc, the co‐localization coefficient was determined using Fiji J software. Images represent at least three independent experiments. Data are means ± SD (*n* = 3 independent experiments, **p < 0.01).

Mitochondrial ribosomes are associated with IMM and mitochondrial matrix in metabolically healthy mitochondria. However, during Drp1/Pink1/Parkin‐mediated fission, membrane reorganization occurs between IMM and OMM (Li et al., 2021; Liu et al., 2021; Nagashima et al., 2020). We hypothesize that mitochondrial membrane rearrangements are critical in recruiting the p17/PERMIT‐CerS1 complex to mitochondria. Mislocalized mitoribosomes to the OMM are essential for this process because they are recognized by p17/PERMIT through its ribosomal protein homology. Our data showed that SoSe induced the OMM localization of CerS1 in UM‐SCC‐1A cells (Figure 1a,b). ICT1, a mitoribosomal protein, was also found in the isolated OMM fractions in response to SoSe (Figure 1a,c). These data were consistent with the mislocalization of mitochondrial proteins Bcl‐2 (OMM protein), Cox‐IV, TIM23 (IMM proteins), Cox‐17, succinate dehydrogenase (SDHA), Cytochrome C (intermembrane space, IMS, proteins), and Bcl‐Xl (transmembrane protein) (Figure S1a–i), supporting of membrane rearrangements, in response to SoSe. Next, we determined the effects of SoSe‐mediated mitophagy on the OMM localization of ICT1 (Richter et al., 2010) and NUBPL (Friederich et al., 2020) (IMM proteins) with/without proteinase K (PK) exposure. PK degrades proteins localized to the OMM selectively in vitro (Bisaccia et al., 1994). Our data demonstrated that SoSe induced OMM localization of ICT1 and NUBPL from the IMM, prompting degradation of the proteins by PK treatment, compared to controls (‐SoSe) (Figure 1d–f). The knockdown of Drp1 prevented OMM localization of ICT1 and NUBLP in response to SoSe (Figure 1d–f). In co‐IP studies, SoSe increased ICT1 and WT‐p17/PERMIT association but not ICT1 and mutant p17/PERMIT (RYE‐AAA, residues 28–30), which cannot be localized to the OMM (Figure 1g,h). Importantly, the knockdown of ICT1 (Figure 1j) using siRNA almost completely inhibited mitochondrial translocation of CerS1 (Figure 1k–m) and mitophagy. Our data demonstrated increased CerS1 activity (Spassieva et al., 2006) in isolated mitochondrial fractions from neuronal cells in response to SoSe (Figure S2a–j). These data also show that CerS1 activity is detectable in mitochondrial extracts, which seems to depend on mitochondrial sphingosine to generate C18‐ceramide, possibly through the salvage pathway. We also observed that the mitochondrial extracts do not exhibit a desaturase function as using dihydrosphingosine as a substrate generated only dihydro‐C18‐ceramide but not C18‐ceramide in vitro (Figure S2a–j).

Membrane rearrangements resulting in mislocalization of mitochondrial proteins in response to SoSe at 0, 1.5, and 3 h exposures in UM‐SCC‐1 cells were also detected by transmission electron microscopy and gold‐labeled anti‐TOM20 antibody (Figure S3a). To define the role of p17/PERMIT‐ICT1 association in CerS1's mitochondrial trafficking, we generated a mutant of p17/PERMIT with the conversion of Phe‐Leu‐Arg‐Asn (residues 39–42) to Ala. These residues are predicted to be involved in ribosome binding (RB) based on the homology of ribosomal L1 protein's 23S rRNA recognition motif (Figure S3b). We then expressed WT‐p17/PERMIT or mutant p17/PERMIT^RBmut^ in cells stably transfected with shRNAs that target endogenous p17/PERMIT. Knockdown of p17/PERMIT prevented mitochondrial CerS1 localization compared to SCR‐shRNA‐transfected controls (Figure S3c,d). While ectopic expression of WT‐p17/PERMIT restored CerS1's mitochondrial localization in p17/PERMIT knockdown cells, mutant p17/PERMIT^RBmut^ failed to mediate this process (Figure S3c,d). In co‐IP studies, WT and mutant‐p17/PERMIT^RBmut^ are associated with CerS1 at comparable levels (Figure S3e,f). While WT‐p17/PERMIT is associated with ICT1, the interaction between ICT1 and mutant p17/PERMIT^RBmut^ was prevented in the absence of endogenous p17/PERMIT in response to SoSe (Figure S3e,g).

Moreover, our data also demonstrated that the activation of Drp1 and mitochondrial fission occur before the recognition of mitoribosomes/ICT by p17/PERMIT at the OMM to induce CerS1/C18‐ceramide‐dependent mitophagy, which appears to be independent of Mul1‐ULK1 or p62/SOSTM1 (Figure S4a–h). These data suggest that mitochondrial membrane rearrangements, resulting in the displacement of ribosomes and ribosomal proteins, such as ICT1, on the OMM, play critical roles in the recruitment of CerS1 to the OMM by p17/PERMIT via its 39‐Phe‐Leu‐Arg‐Asn‐42 residues to induce mitophagy.

### 2.2 Genetic loss of p17/PERMIT disrupts ceramide‐dependent mitophagy

**FIGURE 2:**
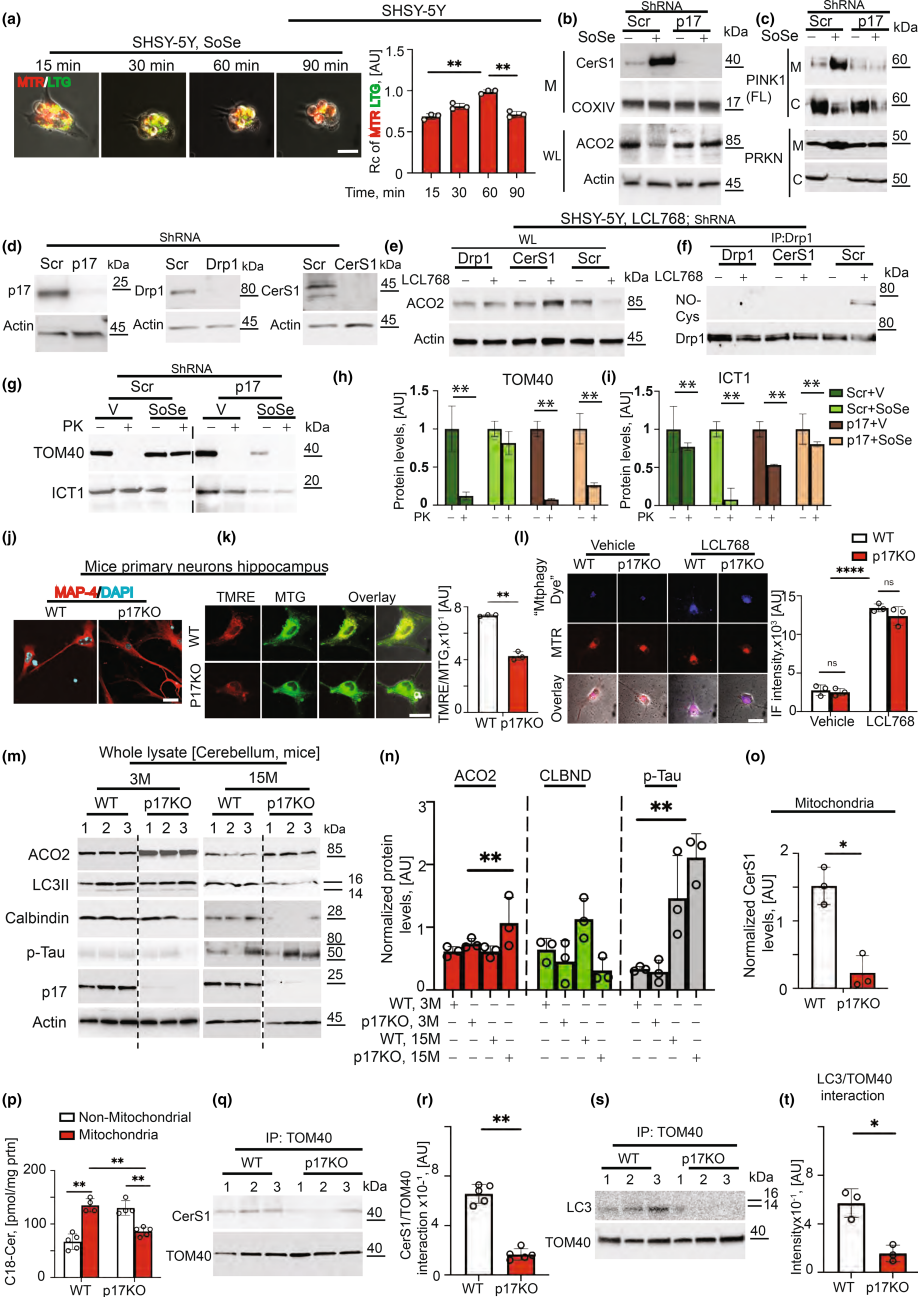
Silencing or genetic loss of p17/PERMIT inhibits SoSe‐induced mitophagy. (a) (Left) Live cell‐confocal imaging (overlay) of SHSY‐5Y‐differentiated neuronal cells treated with SoSe (10 µM) and stained with Mitotracker Red and Lysotracker Green. Right, Quantification of the left. Data are means ± SD (*n* = 3 independent experiments, **p < 0.01). (b) Markers of SoSe‐induced mitophagy shown in mitochondria (M) and whole lysate (WL) of scrambled control and p17 knocked down SHSY‐5Y cells treated for 3 h with vehicle (−) or 10 µM of SoSe (+). COXIV and Actin were used as loading controls. (c) Levels of PINK1 (top panel) and PARKIN (PRKN) (lower panel) in mitochondrial (M) and non‐mitochondrial (NM) fractions of scrambled control and p17 knocked down SHSY‐5Y cells treated for 3 h with vehicle (−) or 10 µM of SoSe (+). (d) Levels of p17 (left), Drp1 (middle) and CerS1 (right) in scrambled control and p17 (left), Drp1 (middle) and CerS1 (right) knocked down SHSY‐5Y neurons. (e) Levels of ACO2 in cells treated with Vehicle (−) or LCL768 (+). Actin demonstrates equal protein loading. (f) Levels of Drp1‐Cys nitrosylation in cells from (e), measured by co‐IP using an antibody for Drp1 and probed against Cys‐NO. (g) Topological analysis of the mitochondrial proteins using PK digestion. TOM40 (top) and ICT1 (bottom) proteins in mitochondria isolated from SCR control and Shp17 SHSY‐5Y cells treated with vehicle or SoSe. (h and i) Quantification of (g). The proteins extracted from PK‐treated samples were normalized by the corresponding levels of untreated cells. Data are means ± SD (*n* = 3 independent experiments, **p < 0.01). (j) Confocal images of primary neurons isolated from the hippocampus of 12 months old WT and p17KO animals stained against MAP‐4 (neuronal marker, red) and DAPI (blue). (k) Detection of mitochondrial membrane potential by staining live primary hippocampal neurons with TMRE (red) and mitotracker (MTG, green). Yellow indicates healthy mitochondria with high mitochondrial membrane potential (left). Right, quantification of the left panel. (l) (left panel), Confocal images of live primary neurons from (j), treated with Vehicle or LCL768 stained with “Mtphagy Dye” (blue) and mitotracker (MTR, red). Magenta indicates mitochondria undergoing mitophagy. Right, quantification of the left. Data are means ± SD (*n* = 3 independent experiments, ****p < 0.0001). (m) Protein (ACO2, LC3, Calbindin, p‐Tau, p17) levels in the whole lysate of cerebellums isolated from 3 (left) to 15 (right) months old WT and p17KO animals. Actin was used as a loading marker. (n) Quantification of the m was done by Image G software. Data are means ± SD (*n* = 3, nsp > 0.05, **p < 0.01). (o) CerS1 protein in mitochondria isolated from cerebellums of animals from (m), COXIV was used as a loading marker. (p) Quantification of (o). Data means ± SD (*n* = 3, *p < 0.05). (q) Levels of C18‐Ceramide measured in mitochondrial (M) and non‐mitochondrial (NM) fractions isolated from cerebellums of 15 months old WT and p17KO animals measured by lipid profiling. (r) Levels of CerS1 and TOM40 interaction in cerebellums isolated from 15 months old WT and p17KO mice measured by co‐IP. s, Quantification of (r). Data means ± SD (*n* = 3, **p < 0.01). (t) Levels of LC3 and TOM40 interaction in cerebellums isolated from 15 months old WT and p17KO mice measured by co‐IP. (u) Quantification of (t). Data means ± SD (*n* = 3, *p < 0.05).

Because mitophagy maintains a healthy brain and neuronal aging, we examined whether mislocalized mitoribosomes/ICT1 control ceramide‐mediated mitophagy by p17/PERMIT in differentiated SHSY‐5Y cells. The data using live cell imaging demonstrated a time‐dependent lysosome‐guided mitochondria (yellow) degradation (mitophagy) induced by SoSe (Figure 2a). Induction of mitophagy was confirmed in differentiated SYSY‐5Y, stained with mitophagy dye (Mtphagy) or expressed pH sensitive mt‐Keima fluorescent protein targeted to mitochondria (Sun et al., 2017) followed by SoSe exposure (Figure S5a–f). As a positive control for mitophagy, we used an inhibitor of mitochondrial oxidative phosphorylation, carbonyl cyanide m‐chlorophenyl hydrazone (CCCP) (Figure S5g,h).

ShRNA‐mediated p17/PERMIT silencing reduced SoSe‐mediated mitochondrial translocation of CerS1 and degradation of ACO2 (Figure 2b). Knockdown of p17/PERMIT also decreased SoSe‐activated mitochondrial accumulation of PINK1 and PARKIN (Figure 2c). The C18‐ceramide analog drug targeting damaged mitochondria (Figure S5i,j), LCL768, induced mitophagy measured by Mtphagy dye (Figure S5k,l) which was inhibited by shRNA‐mediated knockdown of Drp1 or CerS1 (Figure 2d–f). Silencing of p17/PERMIT hindered SoSe‐dependent rearrangements between OMM and IMM in SHSY‐5Y cells (Figure 2g–i). We then performed similar studies using primary neuronal cells isolated from the hippocampus of WT and p17/PERMIT−/− (p17KO) C57BL/6 mice, 15‐month‐old (Oleinik et al., 2019), confirmed by immunofluorescence with an anti‐MAP4 antibody (neuronal cell marker) and DAPI staining (Figure 2j). Mitochondrial potential detected by tetramethylrhodamine ethyl ester (TMRE) staining of the primary neurons (Figure 2k) demonstrated highly polarized healthy mitochondria, which was impaired in p17/PERMIT−/− neurons (Figure 2k). Primary neuronal cells isolated from both WT and p17/PERMIT−/− mice demonstrated a strong mitophagic response to LCL768 (Figure 2l). These data suggest that mislocalized mitoribosomes guide p17/PERMIT‐ceramide‐dependent mitophagy in neuronal cells. Molecular knockdown or genetic loss of p17/PERMIT inhibits CerS1/ceramide‐mediated mitophagy in response to SoSe, but not to LCL768 exposure, resulting in the neuronal accumulation of damaged mitochondria.

Then, we measured the abundance of several mitophagic protein markers in the brain (cerebellum) tissues obtained from p17/PERMIT−/− and WT mice at 3 versus 15 months. Higher ACO2 and decreased LC3 lipidation (autophagic marker) in p17/PERMIT−/− compared to WT mice were detected in 15 months mice (Figure 2m,n). In p17/PERMIT−/− mice (15 months), calbindin (Purkinje cell marker) almost disappeared compared to WT counterparts (Figure 2m,n). We detected higher levels of CerS1 and C18‐ceramide in the cerebellum mitochondria of the WT mice compared to p17/PERMIT−/− mice (15 months) (Figure 2o–q; Figure S6a,b). In the cerebellum of the p17/PERMIT−/− mice, CerS1‐TOM40 and LC3‐TOM40 associations (co‐IPs) were ~50% lower than in control mice (Figure 2r–u; Figure S6b). These data demonstrate the importance of the p17/PERMIT/CerS1/C18‐ceramide axis to mediate ceramide‐dependent mitophagy for maintaining mitochondrial metabolism/homeostasis during healthy aging in WT animals, which is altered in the brain tissues (primarily in the cerebellum) of 15 months p17/PERMIT−/− mice.

### 2.3 Ceramide‐mediated mitophagy in the brain tissues induced by acute SoSe exposure alters mitochondrial metabolism in vivo

We then evaluated the effect of SoSe on the oxidative phosphorylation. We treated SHSY‐5Y cells with 10 mM of SoSe for the indicated periods (Figure S6c,d) and measured fluorescence produced by MitoSox red oxidized by mitochondrial superoxide. The earliest signal was observed at 45 min, while the maximum of superoxide was produced at 60 min of SoSe treatment (Figure S6c,d). These results were consistent with the measurements of mitochondrial oxidative phosphorylation using the oxygen consumption rate (OCR) and ATP production in the vehicle and SoSe (10 µM, 3 h) treated SHSY‐5Y cells (Figure 6e,f). These data indicate that SoSe induces oxidative stress by uncoupling mitochondrial membranes and generating ROS, inhibiting mitochondrial oxidative phosphorylation.

**FIGURE 3:**
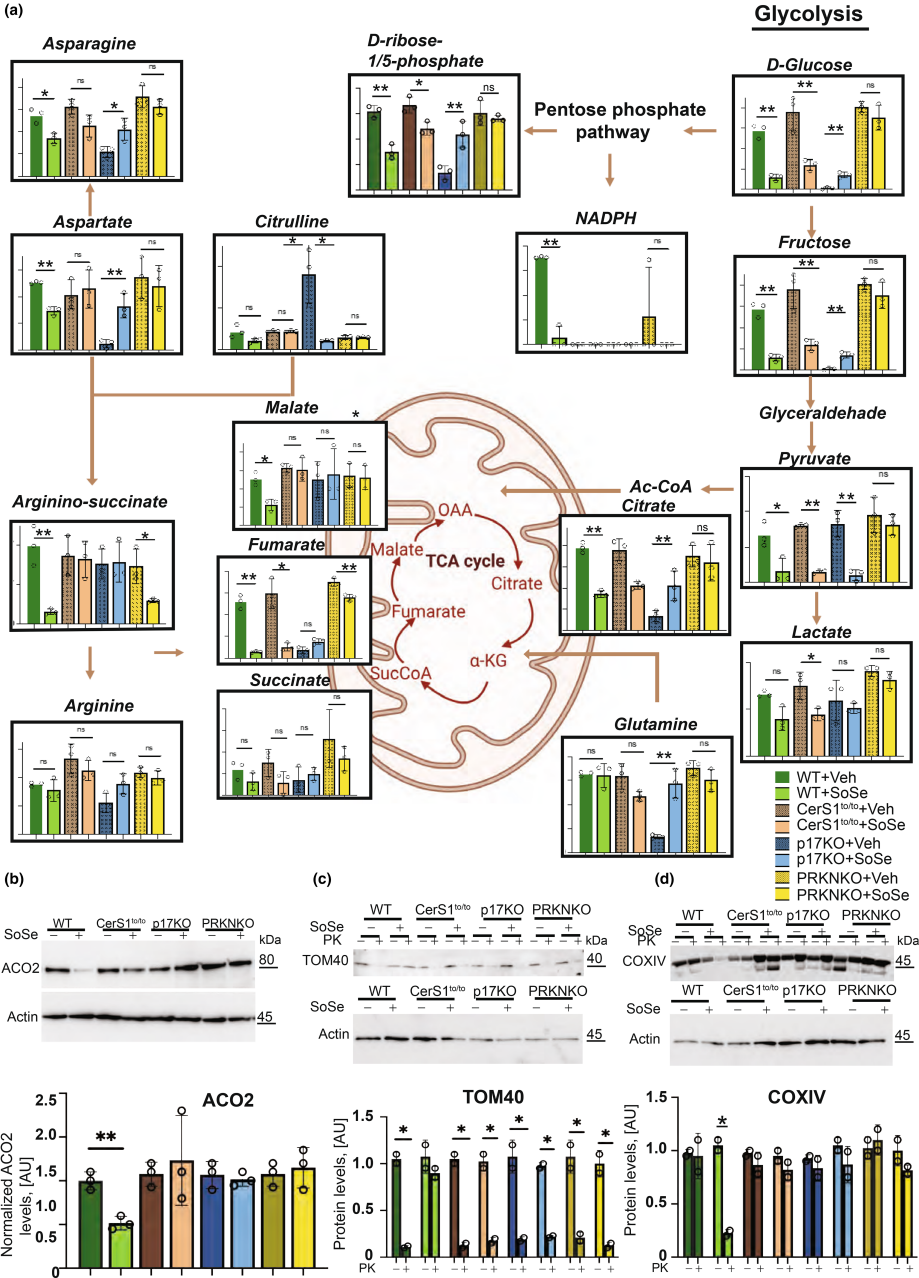
Genetic loss of CerS1, p17/PERMIT, and PARKIN abrogates SoSe‐induced mitophagy response and mitochondrial metabolism in vivo. (a) Metabolomics of the brain (cerebellum) tissues extracted from WT, CerS1to/to, p17KO, and PARKIN KO mice. Levels of metabolites in the cerebellum of WT, CerSto/to, P17KO, and PARKIN KO animals treated either with vehicle or SoSe (1 mg/kg, 3 h). (b) Representative Western blot analysis of ACO2 levels in the brain tissues of WT, CerS1to/to, and PARKIN KO. (c) Quantification of ACO2 levels in brain lysates of mice from (b). Data are means ± SD (*n* = 3 independent experiments, **p < 0.01). Topological analysis of the TOM40 (d) and COXIV (f) proteins by digestion with PK in mitochondria isolated from brain tissues of animals from (a). (e–g) are the quantification of (d) and (f). Data are means ± SD (*n* = 3 independent experiments, *p < 0.05).

Next, we performed metabolomics using mass spectrometry to explore the metabolic alterations of decreased ceramide‐dependent mitophagy in the brain tissue (cerebellum). We exposed WT versus CerS1to/to, p17/PERMIT (p17)−/− or PARKIN (PRKN)−/− knockout mice (3 months) to acute SoSe treatment (1 mg/kg for 3 h) (Figure 3a). WT‐mice exposed to SoSe exhibited decreased fumarate/malate/argininosuccinate/citrate, aspartate, asparagine, and pyruvate, without affecting glutamine or arginine levels in the brain tissues (Figure 3a). In the brain (cerebellum) tissues isolated from the CerS1to/to and p17/PERMIT−/− mice, malate, argininosuccinate, aspartate, and asparagine levels were maintained in response to SoSe compared to vehicle‐treated controls (Figure 3a). Similarly, levels of pyruvate, fumarate, malate, acetyl‐CoA, aspartate, and asparagine (but not argininosuccinate) were maintained in the cerebellum of the PARKIN−/− mice in response to SoSe (Figure 3a). The mitophagy induction in these tissues in response to SoSe was confirmed by decreased ACO2 protein levels and mislocalized TOM20 or COXIV compared to vehicle‐treated controls (Figure 3b–g). These data were consistent with the reduction of argininosuccinate, malate, and fumarate in an age‐dependent manner in the cerebellum isolated from 25 months compared to 2.5 or 12 months WT mice. However, argininosuccinate, malate, and fumarate of p17/PERMIT−/− mice cerebellum at 2.5, 12, or 15 months were much higher than their WT counterparts without any age‐dependent reduction (Figure S7a). These data coincided with an overall decrease in mitochondrial C18‐ceramide accumulation in the brain tissues of 2.5, 12, and 15–25 months p17/PERMIT−/− compared to WT mice (Figure S7b). Elevated non‐mitochondrial C18‐ceramide was detected in 15/25 months WT and p17/PERMIT−/− mice cerebellum tissue samples (Figure S7b). These data support that CerS1/PARKIN‐dependent mitophagy alters cellular metabolism by inhibiting the TCA cycle, especially reducing malate/fumarate metabolism in the brain (cerebellum) in response to aging stress or acute ceramide‐dependent mitophagy induction by SoSe.

### 2.4 Reconstitution of malate or fumarate attenuates ceramide‐mediated mitophagy in vivo

We exposed WT mice (C57BL/6) to SoSe acutely (1 mg/kg for 3 h) in the absence/presence of exogenous malate or fumarate to understand their roles in the regulation of ceramide‐mediated mitophagy in vivo. Metabolomics studies showed that administration of malate (MA) or fumarate (FA) restored SoSe‐dependent depletion of argininosuccinate, fumarate (and to lesser extent malate) without any effects on reduced levels of NADPH, asparagine, or aspartate (Figure S8a). We also observed that SoSe exposure decreased glucose, d‐ribosoe‐1‐5‐phosphate, fructose, pyruvate, and lactate levels in the brain tissues compared to controls (Figure S8a). Fumarate or malate reconstitution maintained glucose, d‐ribose‐1‐5‐phosphate, fructose, pyruvate, and lactate levels in the brain tissues (cerebellum) of Y WT mice (Figure S8a).

Furthermore, we confirmed the process of SoSe‐mediated mitophagy in the brain tissues by reduced ACO2 (aconitase 2) and ASL (argininosuccinate lyase), but not CS (citrate synthase), via Western blotting, which was reversed by administration of exogenous MA or FA (Figure S8b–d). Our data demonstrated that FA or MA administration prevented mislocalization of mitoribosomes/ICT1 or VDAC due to OMM in response to SoSe in the cerebellum of these mice compared to controls (Figure S8e,f). These data suggest that exogenous FA or MA attenuates acute mitophagy in the brain (primarily in the cerebellum) in response to SoSe by recovering fumarate/malate depletion in vivo.

### 2.5 Chronic low‐dose SoSe exposure induces mild mitophagy and improves aging‐dependent sensorimotor deficits in WT mice

Based on our lipidomics and metabolomics studies, we hypothesize that restoration of steady‐state mitophagy would prevent the accumulation of damaged mitochondria in neurons (Irazoki et al., 2022; Navarro et al., 2008) and improve healthy brain aging. To test this hypothesis, we exposed young (Y, 2.5 months) and middle‐aged and aged (A, 15–25 months) to low concentrations (0.5 mg/kg for 5 days/week) of SoSe chronically for 28 days. We then evaluated the effects of SoSe on mice's cognitive and sensorimotor behaviors using Y‐maze and accelerating rotarod studies. Although we did not observe any significant impact of SoSe on learning and memory‐related behavior of Y or A mice, SoSe improved the sensorimotor deficits in A mice (Figure S9a,b). Improved sensorimotor functions of A mice by SoSe were consistent with the increased mitochondrial accumulation of C18‐ceramide and elevated ceramide‐TOM20 association compared to vehicle‐treated controls (Figure S9c–e). Chronic SoSe treatment did not affect argininosuccinate/malate/fumarate metabolism in Y versus A cerebellum compared to vehicle‐treated controls. However, SoSe treatment slightly improved D‐glucose, pyruvate, and lactate levels in A mice's brain (cerebellum) tissues compared to vehicle‐treated controls isolated from Y mice (Figure S9f). Thus, these data suggest that inducing mild ceramide‐mediated mitophagy using chronic low‐dose SoSe treatment alleviates aging‐dependent sensorimotor defects by promoting healthy mitophagy and mitochondrial metabolism.

### 2.6 The behavioral assessment of 15 months p17/PERMIT−/− mice demonstrates age‐dependent progressive motor‐neuron deficiency

**FIGURE 4:**
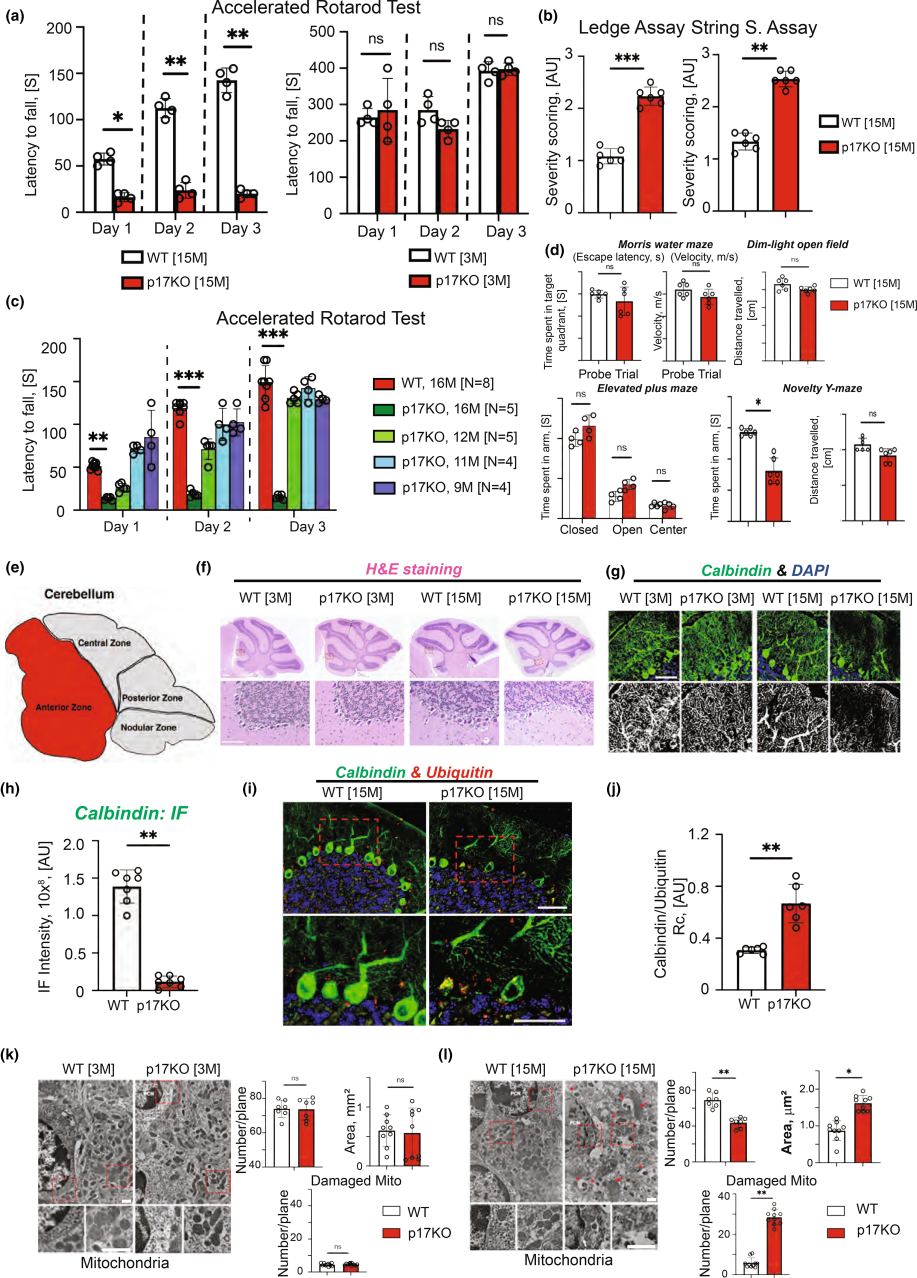
The genetic loss of p17/PERMIT inhibits motor‐neuronal functions in aging mice. (a) Evaluation of 15 (left panel) and 3 months mice motor‐neuron functions by accelerated rotarod test. *Latency to fall* (the time mouse spent on the rotating rod with increasing velocity) of WT and p17KO during accelerated rotarod task for 3 consecutive days. Repeated‐measures two‐way ANOVA was conducted to examine the main effect of genotype on each day (*p* values indicated). *p < 0.05, **p < 0.01 by *t* test with Bonferroni correction. *p < 0.05 by Mann–Whitney test with Bonferroni correction (number of comparisons was 10 for latency to fall). *n* = 12 mice per genotype. Error bars represent SEM. (b) Analysis of coordination and strength by Composite Phenotype Scoring. This scoring was based on the ledge (left) and string (right) assays. Repeated‐measures two‐way ANOVA was conducted to examine the main effect of genotype. *p < 0.05, **p < 0.01, ***p < 0.001 (*n* = 12 mice per genotype). Error bars represent SEM. (c) p17KO animals' motor‐neuron deficit is age‐related. An accelerated rotarod test assessed the mice's coordination. (d) Evaluation of learning and memory by Morris water maze, dim‐light open field, elevated plus maze, and novelty Y‐maze tests. *p* values are indicated (nsp > 0.05). (e) Schematics of the mouse cerebellum's zones. (f) H&E staining of the 3‐ and 15‐month‐old WT and p17KO animals' cerebellum. Insets demonstrate almost complete loss of Purkinje cells in the cerebellum's anterior zone. (g) Upper panels contain confocal microphotographs of the cerebellum of animals from (f) stained against Calbindin, Purkinje marker (green), and DAPI (blue). Lower panels contain insets, as marked in upper panels, with higher magnification. (h) Confocal images of cerebellums of 15 months WT and p17KO animals stained against ubiquitin, calbindin, and DAPI. (i and j) Quantification of (h). For the fluorescence image quantification, Image G software was used (**p < 0.01), *n* for the WT group is 7, and n for p17KO is 6. Error bars represent SEM. (k) Left panel, TEM micrographs of cerebellums of the 3 months WT (left) and p17KO (right) animals. Lower panels are insets from the top with higher magnification. Scale bars, 2 m. Left top, quantification of mitochondria number per plane from TEM on the left. *p* value is indicated nsp = 0.05 (*n* = 7 mice/images per genotype). Error bars represent SEM. Right top, the average area of the mitochondria from TEM is on the left. *p* value is indicated nsp = 0.05 (*n* = 7 mice per genotype). Error bars represent SEM. Bottom, quantification of damaged mitochondria. *p* value is indicated nsp = 0.05 (*n* = 7 mice per genotype). Error bars represent SEM. (l) Left panel, TEM microphotographs of cerebellums of the 15 months WT (left) and p17KO (right) animals. Lower panels are insets from the top with higher magnification. Scale bars, 2 m. Left top, quantification of the mitochondria from EM on the left. *p* value is indicated **p < 0.01 (*n* = 7 mice per genotype). Error bars represent SEM. Right top, the average area of the mitochondria from EM is on the left. **p < 0.01 (*n* = 9 mice per genotype). Bottom, quantification of damaged mitochondria. *p* value is indicated **p < 0.01 (*n* = 7 mice per genotype). Error bars represent SEM.

We explored the effects of impaired mitophagy on cognitive processes and behavioral reactions in p17/PERMIT−/− compared to WT mice regarding aging stress. In the accelerated rotarod test, the latency (riding) time was significantly reduced in 15, but not in 3 months p17/PERMIT−/− compared to age‐matched WT animals (Figure 4a; Video S1), indicating reduced motor coordination of 15 months p17/PERMIT−/− mice. Also, 15 months p17/PERMIT−/− mice performance in the ledge and string tests, which assess coordination and strength, was considerably lower than age‐matched WT counterparts (Figure 4b). Figure 4c shows that sensorimotor defects are age‐dependent, showing deficits at 15–16 months in p17/PERMIT−/− male mice. We did not observe significant changes in cognitive functions, including spatial learning and memory (Figure 4d), or anxiety and hyperactivity (Figure 4d) in 15 months p17/PERMIT−/− compared to age‐matched WT mice. The Novelty Y‐maze test, assessing short‐term memory, was the only exception and showed slightly lower performance in 15 months old p17/PERMIT−/− compared to age‐matched control WT mice (Figure 4d). These data demonstrate that genetic loss of p17/PERMIT results mainly in sensorimotor defects in an age‐dependent manner starting at around 15 months in primarily male p17/PERMIT−/− mice.

### 2.7 Sensorimotor deficiency in p17/PERMIT−/− mice was accompanied by aging‐dependent cerebellar Purkinje cell degeneration and neuronal loss

H&E and immunofluorescence calbindin (a marker of Purkinje cells) staining demonstrated a significant neuronal loss in the Anterior Zone of the 15 months p17/PERMIT−/− mice that was accompanied by vacuolation and dissolution of the normal cerebellum structure in WT animals (Figure 4f–h). Confocal microscopy of the p17/PERMIT−/− cerebellum revealed strong co‐localization of ubiquitin‐positive inclusions and calbindin staining in Purkinje cells, signifying ubiquitin involvement in their deterioration (Figure 4i,j). The ultrastructure of the WT (3 or 15 months) cerebellum examined by TEM showed a typical appearance of intact mitochondria with abundant and well‐organized cristae (Figure 4k,l, left panels). The mitochondria's shape and size were highly variable, consistent with continuous fission and fusion required for cellular homeostasis (Figure 4k,l, left panels). By contrast, the cerebellum collected from 15 months p17/PERMIT−/− mice, but not 3 months, contained larger mitochondria (size) and more damaged than WT controls (Figure 4k,l, right panels) with disarranged membranes. These data suggest that sensorimotor defects of 15 months p17/PERMIT−/− mice are associated with the accumulation of damaged mitochondria in the cerebellum.

### 2.8 Ceramide analog drug, LCL768, restores mitophagy and alleviates aging‐dependent sensorimotor deficiency in p17/PERMIT−/− mice

**FIGURE 5:**
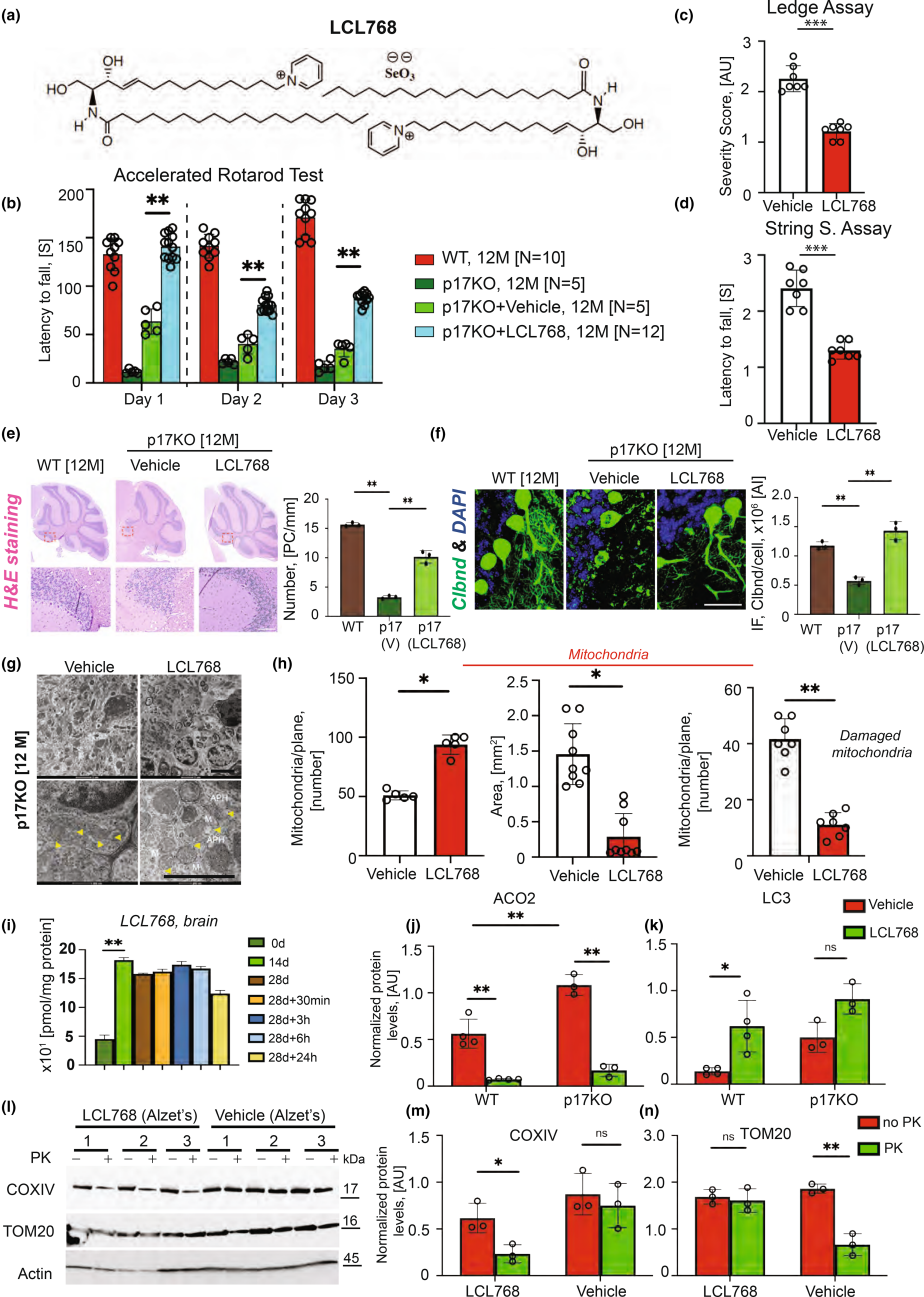
Ceramide analog LCL768 alleviates motor neuron deficiency in 15 months p17/PERMIT−/− (KO) mice by inducing mitophagy. (a) The chemical formula of LCL768, a sphingolipid‐based selenium compound. b, Evaluation of 12 months mice’ motor‐neuron functions by accelerated rotarod test with vehicle (Alzet pump, 28 days) or LCL768 (Alzet pump, 0.1 mg/kg/day for 28 days) during accelerated rotarod task for 3 consecutive days. *p < 0.05, **p < 0.01 by *t* test with Bonferroni correction (*n* = 12 mice per genotype). Error bars represent SEM. (c and d) Analysis of animals' coordination and strength by Composing Phenotype Scoring. A detailed description of the method is in the legend of (b). The average composite score for each p17KO‐vehicle and p17KO‐LCL768 was calculated. Repeated‐measures two‐way ANOVA was conducted to examine the main effect of genotype (*p* values indicated). **p < 0.01 (*n* = 7 mice per genotype). Error bars represent SEM. (e) (left panel), H&E staining of the 12‐month WT and p17KO animals' cerebellum treated with Vehicle or LCL768. Insets demonstrate loss of Purkinje cells in the cerebellum's anterior zone of p17KO and restoration of the cells in treated mice with LCL768; right panel, quantification of the left. (f) Confocal microphotographs of the animals' cerebellums from (e) stained against Calbindin, Purkinje marker (green), and DAPI (blue). (g) TEM micrographs of cerebellums of the 12 months p17KO mice treated with vehicle (left) or LCL768 (right). Lower panels are insets from the top with higher magnification. Scale bars, 2 nm. (h) Left, Quantification of mitochondria number from (g). *p* value is indicated nsp = 0.05, *p < 0.05 (*n* = 4 mice from vehicle‐treated group and *n* = 5 mice from LCL768 treated group). Error bars represent SEM. Middle, the average area of the mitochondria from (g). *p* value is indicated nsp = 0.05, *p < 0.05 (*n* = 9 mice per genotype). Right, Quantification of damaged mitochondria from (g). *p* value, nsp = 0.05, *p < 0.05 (*n* = 7 mice per genotype). (i) Levels of LCL768 in the brain of p17KO mice implanted with Alzet pumps during indicated periods (28 days, +30 min, and 3, 6, or 24 h), measured by mass spectrometry. *p < 0.05, **p < 0.01 (*n* = 3 mice per group). Error bars represent SEM. (j) ACO2 levels in the cerebellum of animals from (b). *p < 0.05, **p < 0.01 (at least 4 mice have been used per group). Error bars represent SEM. (k) LC3 levels in the cerebellum of animals from (b). *p < 0.05 (at least 4 mice have been used per group). Error bars represent SEM. (l) Topological analysis of the COXIV and TOM20 proteins by digestion with PK in mitochondria isolated from brain tissues of animals from (b). (m and n) Quantification of (l). Data are means ± SD (*n* = 3 animals per group, *p < 0.05).

To overcome defects of ceramide‐mediated mitophagy, we designed a ceramide analog drug, LCL768, to accumulate in damaged mitochondria (Figure 5a). To investigate the ability of LCL768 to induce mitophagy in vivo, we treated the animals with 3.0 mg/kg of the compound using tail vein injection to determine its accumulation in various tissues by lipidomics. LCL768 was not detected in the brain (Figure S10a). Therefore, to deliver LCL768 directly into the cerebellum, we surgically implanted Alzet osmotic pumps loaded with LCL768 (0.1 mg/kg/day) or vehicle into the right lateral ventricle of the 12 months WT and p17/PERMIT−/− mice. Following 28 days of LCL768 exposure, mice were subjected to a battery of behavioral tests to evaluate the functioning of the sensory‐motor apparatus (Figure 5b–d). Data showed that LCL768‐treated p17/PERMIT−/− mice demonstrated significant improvement in motor neuron functions (Figure 5b–d). LCL768 inhibited Purkinje cell degeneration in p17/PERMIT−/− mice (15 months) (Figure 5e,f). TME showed an increased number of smaller and well‐organized mitochondria in the cerebellums of the LCL768‐treated p17/PERMIT−/− (p17KO) mice compared to vehicle‐treated controls (Figure 5g,h).

To evaluate LCL768 effects on lipid metabolism, mice were sacrificed at 0, 14, and 28 days after pump implantation, and ceramide profiles were analyzed in the cerebellum (Figure 5i). Data demonstrated that LCL768 levels in the cerebellum reached a plateau 14 days of treatment and stayed at comparable levels for at least 28 days (Figure S10b,c). LCL768 selectively induced the generation of endogenous C18‐ceramide in the total lysate and mitochondria (Figure S10d). LCL768 restored mitophagy in the cerebellum of p17/PERMIT−/− mice (Figure 5j,k; Figure S10e–n), concomitant with improved sensorimotor functions.

**FIGURE 6:**
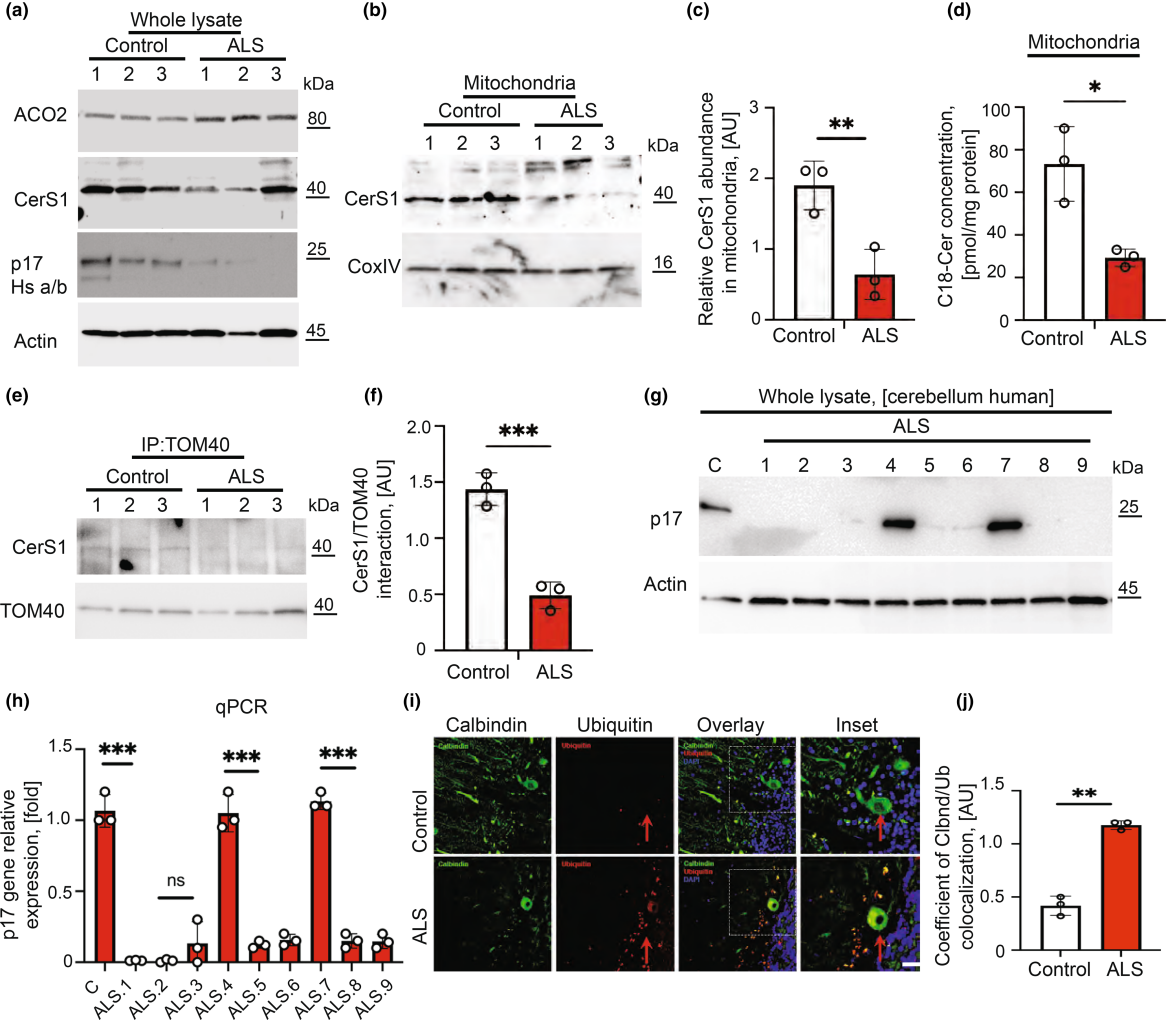
Detection of p17/PERMIT/CerS1/ceramide‐dependent mitophagy in the brain tissues of healthy donors versus ALS patients. (a) Levels of ACO2, CerS1, p17 in the whole lysates brain tissues from control and ALS individuals. Actin is used as a loading control. (b) CerS1 in mitochondria isolated from cerebellums from (a). COXIV was used as a loading control. (c) quantification of (b). Data are means ± SD (*n* = 3, **p < 0.01). (d) Levels of C18‐ceramide were measured in mitochondrial fractions, isolated from the cerebellum tissues of control individuals and ALS patients by lipid profiling. (e) Levels of CerS1 and TOM40 interaction in cerebellums isolated from control and ALS patients were measured by co‐IP. (f) Quantification of (e). Data are means ± SD (*n* = 3, ***p < 0.001). (g) Levels of p17 in whole lysates isolated from the cerebellum of control and ALS patients. Actin is used as a loading control. (h) Levels of p17 gene relative expression in control and ALS human cerebellums. Data are means ± SD (*n* = 3, ***p < 0.001). (i) Confocal images of the cerebellums isolated from human age‐matched control and ALS patients and stained with calbindin (green), ubiquitin (red), and DAPI (blue). The yellow image shows the co‐localization of calbindin and ubiquitin. Arrows point at Purkinje cells. (j) Quantification of (i). Data are means ± SD (*n* = 3, **p < 0.01).

Because aging‐dependent sensorimotor defects (primarily in aging males) and Purkinje cell degeneration detected in p17/PERMIT−/− mice were like those seen in patients with ALS, we measured ACO2, CerS1, and p17/PERMIT in mitochondria isolated from the verified brain tissues of patients with ALS versus non‐ALS donors (Tables 1 and 2). The data showed that while there were no significant changes in the ACO2, CerS1, and LC3‐II levels, there was a substantial loss of p17/PERMIT expression in whole‐cell brain (cerebellum) extracts (Figure 6a). This was accompanied by a considerable reduction of CerS1 and C18‐ceramides accumulation in cerebellum mitochondria of ALS brains compared to controls (Figure 6b–d). Compared to non‐ALS donors, the association between CerS1‐TOM40 in the cerebellum tissues isolated from ALS patients was decreased (Figure 6e,f). Seven of nine sporadic ALS patients showed decreased p17/PERMIT at the protein and mRNA levels compared to controls, consistent with defective ceramide‐mediated mitophagy (Figure 6g,h). Finally, the degree of proteasomal degradation (apoptosis marker) was assessed in Purkinje cells of control and ALS individuals. As evident from the solid yellow signal observed in the ALS neurons (Figure 6i,j), the apoptotic pathway was more active in ALS's cerebellum than in control donors. Thus, these data suggest that defects in cerebellum mitophagy in sporadic ALS patients may be partly attributed to alterations of the p17/PERMIT/CerS1‐mitophagy axis. These findings support the clinical relevance of this mechanism in neurodegenerative disorders associated with sensorimotor defects, including ALS.

**TABLE 1:** Human samples and patient demographics information.

ALS donor #	Disease duration^a^	Sex	Age	Ethnicity	ALS	pH^b^	RIN^b^	PMI‐cr^c^	PMI‐frz^d^
090009	84	Male	65	W	SALS	6.53	4.7	10.0	80.0
090010	96	Male	73	W	SALS	6.46	5.7	0.33	54.0
090011	97	Male	81	W	SALS	6.32	4.8	10.0	50.0
090014	50	Male	77	W‐Am Ind	Unknown	5.99	5.4	3.25	24.0
130003	248	Male	74	W	SALS	6.51	6.1	1.08	52.08
AZ160016	29	Male	72	W	SALS	6.86	7.3	0.75	32.0
AZ190011	288	Male	87	W	SALS	6.28	6.3	1.00	42.0
AZ190014	36	Male	60	W	SALS	6.59	5.1	Unknown	30.64
AZ200006	Unknown	Male	78	Unknown	SALS	6.16	6.2	Unknown	28.09
AZ200017	24	Male	78	W	SALS	6.17	5.8	1.50	48.50

^a^ Time of recorded symptom onset in months.

^b^ Indicators of tissue quality: RNA Integrity Number (RIN), tissue pH (indicator of subject agonal state). Data derived from single brain region (occipital lobe). Brain tissue quality indicators have been discussed in the scientific literature and most closely correlate with subject agonal state prior to death (vs. PMI times).

^c^ PMI‐cr: post‐mortem interval, approximate time from death to body refrigeration (hours).

^d^ PMI‐frz: post‐mortem interval, approximate time from death to the brain to be frozen at ‐80°C.

**TABLE 2:** Human samples and patient demographics information.

Control donor #	Sex	Age	Ethnicity	pH^a^	RIN^b^	PMI‐cr^c^	PMI‐frz^d^
090015	M	66	H	6.15	5.7	4.0	83.00
100012	F	81	W	6.70	6.2	Unknown	40.00
100017	M	70	W	6.38	6.4	1.6	56.00
100021	M	78	W	6.68	5.8	4.0	27.4
130013	M	66	W	5.98	5.2	2.92	70.92

^a,b^ Indicators of tissue quality: ^a^pH (indicator of subject agonal state). ^b^RNA Integrity Number (RIN). Data derived from a single brain region (occipital lobe). Brain tissue quality indicators have been discussed in the scientific literature and most closely correlate with the subject agonal state prior to death (versus PMI times).

^c^ PMI‐cr: post‐mortem interval, approximate time from death to body refrigeration (hours).

^d^ PMI‐frz: post‐mortem interval, approximate time from death to the brain to be frozen at‐ 80°C.

## 3 DISCUSSION

Here, we show that during healthy aging, mitophagy induction of stress‐mediated CerS1/C18‐ceramide signaling is orchestrated by rearrangements between OMM and IMM guided by activation of Drp1 and the fission process. When Drp1 is activated by nitrosylation, it loses its ability to interact with p17/PERMIT, leading to Drp1‐triggered fission, mitochondrial membrane rearrangements, and OMM localized ICT1/mitoribosomes that are recognized by the p17/PERMIT to mediate mitochondrial transport of CerS1 to induce mitophagy, leading to the depletion of fumarate/malate in various cells and metabolically active brain tissues (primarily in the cerebellum) in vivo. Moreover, exogenous FA or MA in mice prevented ceramide‐mediated mitophagy and blunted mitochondrial quality control in the brain tissues of mice. Importantly, genetic loss of p17/PERMIT resulted in aging‐dependent sensorimotor deficits in p17/PERMIT−/− mice at 15 months, associated with altered mitophagy or metabolism and accumulation of damaged mitochondria in the brain associated with sensorimotor defects. However, ceramide‐dependent mitophagy was restored through the intracranial delivery of a ceramide analog drug, LCL768, vastly improving sensorimotor functioning of aging p17/PERMIT−/− mice in vivo.

Mitoribosomes are specialized to translate mitochondrial genes that encode protein subunits involved in oxidative phosphorylation (Cruz‐Zaragoza et al., 2021; Greber et al., 2014; Hansen et al., 2018). Mitoribosome defects were associated with poor clinical outcomes in patients with hepatocellular carcinoma (Wu et al., 2018). These data are consistent with the results presented here, demonstrating that p17/PERMIT recognizes mitoribosomes localized to the OMM by Drp1 through p17/PERMIT's 39–41 (FLRN) residues, which are homologous to the ribosome‐protein binding four‐amino acid‐stretch (19–22, FTQS) (Nevskaya et al., 2000).

Earlier studies showed that arginine starvation induces cell death in cancer cells via aspartate exhaustion and mitochondrial damage/dysfunction (Crump et al., 2021). This process was due to the asparagine synthetase (ASNS) induction, which eventually depletes aspartate, disrupting the malate–aspartate shuttle in response to arginine auxotrophy (Cheng et al., 2018). Recent studies showed that fumarate could control epigenetic regulation in various models, supporting the idea that the metabolic intermediates might have additional functions (Frezza et al., 2011; Xiao et al., 2012).

The sensorimotor deficiencies in p17/PERMIT−/− mice were linked to cerebellum Purkinje‐cell deprivation in aging mice, consistent with increased accumulation of damaged mitochondria. These data agree that Purkinje‐cell defects are associated with ataxia, a phenotype seen in CerS1to/to mutant mice expressing a catalytically inactive enzyme (Zhao et al., 2011), leading to reduced C18‐ceramide levels in the brain. Recent reports indicated that age‐associated cerebellar degeneration may contribute to ALS's motor and non‐motor symptoms (Abidi et al., 2021; Bede et al., 2021; Pradat, 2021).

Mitophagy might harm aging T cells (Vaena et al., 2021), leading to immune suppression.

However, in healthy brain tissues, it mediates the elimination of damaged mitochondria, providing mitochondrial quality control and preventing sensorimotor defects, suggesting context‐dependent outcomes, which need to be defined. Also, the clinical significance of p17/PERMIT‐CerS1‐C18‐ceramide‐mediated mitophagy alterations in the development and progression of ALS or other aging‐dependent sensorimotor deficiencies must be determined in a larger cohort of patients. Moreover, the exact mechanism by which LCL768 restores mitophagy, directly or indirectly, by inducing mitochondrial CerS1/C18‐ceramide remains unknown. Nevertheless, our results warrant further studies to test the therapeutic roles and efficacy of LCL768 to induce mitophagy and improve sensorimotor defects in neurodegenerative diseases like ALS.

## 4 MATERIALS AND METHODS

### 4.1 Cell lines and culture conditions

Cells were cultured in Dulbecco's modified Eagle's medium (DMEM) (Cellgro) with 10% fetal bovine serum (FBS) (Atlanta Biologicals) and 1% penicillin and streptomycin (Cellgro).

### 4.2 Plasmids, antibodies, and shRNAs

Details of plasmids, antibodies, and shRNAs are provided in the Key Resources Table.

**Table:** 

Reagent or resource	Source	Identifier
Antibodies		
ICT1	Abcam Cat# ab55259	RRID:AB_2122854
NUBPL	Abcam Cat# ab69150	RRID:AB_2153651
COXIV	Abcam Cat# ab131177	RRID:AB_11154973
Tom20	Santa Cruz Biotechnology Cat# sc‐17764,	RRID:AB_628381
Tom40	Santa Cruz Biotechnology Cat# sc‐365466	
LASS1	Santa Cruz Biotechnology Cat# sc‐65096	N/A
LASS1	My BioSource.com	#MBS7104965
COX17	Santa Cruz Biotechnology Cat# sc‐100521	RRID:AB_2085114
COX4	Cell Signaling Technology Cat# 4850	RRID:AB_2085114
Parkin	Santa Cruz Biotechnology Cat# sc‐32282	RRID:AB_628104
PINK	Santa Cruz Biotechnology Cat# sc‐517353	
LC3B	Cell Signaling Technology Cat# 2775	RRID:AB_915950
ACO2	Cell Signaling Technology Cat# 6922S	RRID:AB_10828218
GFP	Thermo Fisher Scientific Cat# 14‐6774‐63	RRID:AB_468332
PDI	Novus Cat# NB100‐1921	RRID:AB_10001061
Cystein S‐Nitrosylated (SNO‐Cys)	US Biological Life Sciences Cat# C9002‐75	N/A
DLP1	BD Transduction Lab Cat# 611112	N/A
V5	Invitrogen Cat# R96025	N/A
VDAC	Cell Signaling Technology Cat# 4661	
ASL	Abcam Cat# ab97370	
CS	Cell Signaling Technology Cat #14309	
iNOS	Cell Signaling Technology Cat # 13120	
Calbindin	Santa Cruz Biotechnology Cat# sc‐36530	
Ubiquitin	Santa Cruz Biotechnology Cat# sc‐ 8017	
Actin	Sigma‐Aldrich Cat# A2066	RRID:AB_476693
Ceramide	Enzo Life Sciences Cat# ALX‐804‐196	RRID:AB_10541503
p17 (Ribosomal Protein L29 (P‐14)	Santa Cruz Biotechnology Cat# sc‐103166	N/A
p17 (Ribosomal Protein L29)	Abcam Cat# ab88514	RRID:AB_2042834
P17 (Ribosomal Protein L29)	In the house	N/A
BCL2	Cell Signaling	RRID:AB_2228008
TIM23	Proteintech	RRID:AB_615045
SDHA	Cell Signaling	RRID:AB_10707493
Bcl‐Xl	Cell Signaling	RRID:AB_2744528
ULK1	Cell Signaling	RRID:AB_2212518
p‐Ser555‐ULK1	Cell Signaling	RRID:AB_10707365
P62	Cell Signaling	RRID:AB_2800125
Chemicals, peptides and recombinant proteins		
LCL768	In the house	
LCL768‐Cy.5	In the house	
Maleic acid	Sigma Cat# MO375	
Fumaric acid	Sigma Cat# 47910	
Phosphate buffered saline (PBS)	Gibco	10010‐023
Dulbecco phosphate	Corning	12491‐015
Fetal Bovine Serum (FBS)	Atlanta Biologicals	S12495H
Penicillin/Streptomycin	GIBCO	15140‐122
Dulbecco's Modified Eagle Medium (DMEM)	GIBCO	12491‐015
Effectene	Qiagen	301425
Protease Inhibitor	Sigma	P8340
Trypsin/EDTA	Sigma	T4049
Doxycycline Chloride	Sigma	D3072
SIN‐1 Chloride	Cayman Chemical Company	82220
Sodium Selenite	Sigma	S5261
Mtphagy Dye	Dojindo Molecular Technologies, Inc., MT02	MT02
P62‐mediated mitophagy inducer	MedChemExpress	HY‐115576
MitoSox Red	ThermoFisher Scientific	M36009
SBI‐0206965	Stem Cell Technologies	100‐0269
CellTiter‐Glo® Luminescent Cell Viability Assay	Promega	G7570
C17‐sphingosine	Avanti Polar Lipids	860602
C18:0 fatty acyl‐CoA	Avanti Polar Lipids	870718
C17 dihydrosphingosine	Avanti Polar Lipids	860602P
Oligomycin	Sigma	O4876
Rotenone	Sigma	557368
Antimycin A	Sigma	A8674
FCCP	Sigma	C2920
TMRE	Sigma	T669
Experimental models: cell lines		
Human: SHSY‐5Y	ATCC	RRID:CVCL_0023
Human: UM‐SCC‐1A	Dr. T. Carey (Department of Otolaryngology/Head and Neck Surgery, University of Michigan, Flint, MI, USA)	RRID:CVCL_7707
Human: UM‐SCC‐22A	Dr. T. Carey (Department of Otolaryngology/Head and Neck Surgery, University of Michigan, Flint, MI, USA)	RRID:CVCL_7731
Human: UM‐SCC‐22A shDrp1	Ogremen's lab	N/A
Human: UM‐SCC‐22A shp17	Ogretmen's lab	N/A
Experimental models: organisms/strains		
Mouse: FVB/NJ^*WT*^	The Jackson Lab	
Mouse: FVB/NJ^*To/To*^	The Jackson Lab	
Mouse: C57Bl/6*j* ^*WT*^	Charles Rivers	
Mouse: C57Bl/6j P17KO	This paper	
Mouse: PARKIN KO	The Jackson Lab	
Reagents		
DMEM	Cytiva	Cat# SH30243.01
PBS pH 7.4 (1x)	Gibco	Cat# 10010‐023
Fetal bovine serum	Avantor	Cat # 97068‐085
Penicillin‐streptomycin (100x)	Corning	Cat # 30‐002‐CI
PlasmocinTM Prophylactic	InvivoGen	Cat# ant‐mpp
0.05% trypsin‐0.02% EDTA	Corning	Cat# 25‐052‐CI
Trypan Blue solution	MilliporeSigma	Cat# T8154
MTT Cell Proliferation Assay	ATCC	30‐1010K
4% PFA	Boster	Cat# AR1068
10% acetic acid	Fisher Scientific	Cat# 135‐32
Pierce RIPA buffer	Thermo Fisher Scientific	Cat# 89900
DTT	Cell Signaling Technology	Cat# 7016L
Protease Inhibitor Cocktail	Sigma‐Aldrich	Cat# P8340
PMSF Protease Inhibitor	Thermo Fisher Scientific	Cat# 36978
SuperSignal™ West Pico PLUS Chemiluminescent Substrate	Thermo Fisher Scientific	Cat# 34580
Q5 Site‐directed mutagenesis	New England Bio‐Labs	E0554S
Plasmids, primers		
pMD2.G	Addgene	#12259
psPAX2	Addgene	#12260
pEGFP‐Drp1wt	a gift from Dr. S. A. Lipton (Burnham Institute for Medical Research, La Jolla)	
pEGFP‐Drp1K38A(DN)	a gift from Dr. S. A. Lipton (Burnham Institute for Medical Research, La Jolla)	
pEGFP‐Drp1C644A	a gift from Dr. S. A. Lipton (Burnham Institute for Medical Research, La Jolla)	
pEGFPDrp1C644W	Generated in the lab: F5′‐CGG GAA CAG CGA TGG GAG GTT ATT GAA CGA‐3′, R5′‐TCG TTC AAT AAC ATC CCA ATC TCG CTG TTC CCG‐ 3′.	
p17RYE28‐30AAA	Generated in the lab.	Oleinik et al. (2019)
p17Rbmut	Generated in the lab from FLAG‐HA‐pcDNA3.1‐p17wt; 1 Set:F5′‐GGG TGG ACC CCA AGG CCG CGG CGG CCA CGT GCT TTG CCA AG‐3′; R5″‐GTG CTT CTT GGC AAA GCA CGT GGC CGC CGC GGC CTT GGG GTC CAC‐3′; 2 Set: F5′‐CGG TGG ACC CCA AGT TCG CGG CCA ACA CGT GCT TTG CCAAG‐3′; R5′‐GTG CTT CTT GGC AAA GCA CGT GTT GGC CGC GAA CTT GGG GTC‐3′.	
siRNA and Lentiviral clones to express shRNA and		
Drp1	Sigma‐Aldrich	TRCN0000001097
ICT1	Sigma‐Aldrich	TRCN0000159769
RPL29P31 (p17)	Sigma‐Aldrich	TRCN0000184844
Non‐targeting (SCR)	Sigma‐Aldrich	SHC002
Software and algorithms		
Phyre2	Kelley, L.A., et al. (2015). Nat Protoc *10*, 845‐858	
ZDOCK	Pierce, B.G., et al, (2014).Bioinformatics 30, 1771–1773	
PredictProtein	Yachdav, G., et al. (2014). Nucleic Acids Res 42, W337–343	
MitoPro	M. G. Claros (1995) Comput. Applic. Biosci. 11, 441–447	
PRISM	GraphPad Software	Version 9

### 4.3 Immunoprecipitation and Western blotting

Cellular lysates in RIPA buffer containing a protease inhibitor cocktail (Sigma‐Aldrich) were normalized by the total protein level and analyzed by SDS‐PAGE and immunoblotting with corresponding antibodies as described (Oleinik et al., 2019).

### 4.4 Immunofluorescence

Immunofluorescence was performed using a Leica TSC SP2 AOBS TCS confocal microscope, Zeiss 880 LSM NLO with AiryScan, and Olympus FV10i microscope with 543‐ and 488‐nm channels for visualizing red and green fluorescence. Images were taken at ×63 magnification. At least three random fields were selected for images.

### 4.5 Ultra‐structural analysis using TEM

UM‐SCC‐22A cells were fixed in 2% glutaraldehyde (w/v) in 0.1 M cacodylate following the removal of the culture medium. After post‐fixation in 2% (v/v) osmium tetroxide, specimens were embedded in Epson 812. Thin sections were visualized in a JEOL 1010 transmission electron microscope (Oleinik et al., 2019).

### 4.6 Cell fractionation

For mitochondria preparation, Mitochondria Isolation Kit (ab110171, Abcam) was used according to manufacturer instructions. Mitochondria were isolated, and submitochondrial fractionation was performed as described (Jensen et al., 2014).

### 4.7 Analysis of sphingolipids by lipidomics

Lipid extractions and analyses were performed by Lipidomics Shared Resource, Analytical Unit (MUSC) by mass spectrometry, as we described (Oleinik et al., 2019).

### 4.8 Live‐cell imaging

Time series of live cell‐confocal images were collected every 1–2 min for 150 min after adding SoSe with Olympus FluoView FV10i LIV laser scanning confocal microscope at the MUSC Cell and Molecular Imaging Core Facility. Mitophagy was assessed using the Mtphagy dye (MT02, Dojindo Molecular Technologies, Inc.) or pHAGE‐mtKeima expression.

### 4.9 Steady‐state metabolite profiling and targeted metabolic flux analysis

The metabolic profiling was performed at the Metabolomics Core Facility of Feinberg School of Medicine at Northwestern University (Chicago, IL) by LC–MS/MS. Data were analyzed by the MetaboAnalyst V5.0 platform (https://www.metaboanalyst.ca) (Pang et al., 2021). The details of metabolic flux analysis and ATP measurements can be found in Supplementary Materials and Methods.

### 4.10 Oxidative consumption rate measurements

The measurements of OCR and extracellular acidification rate (ECAR) in living cells have been done by real‐time flux analyses using the Seahorse platform as we previously described (Chakraborty et al., 2019; Sentelle et al., 2012) using the Seahorse XF96 analyzer (Agilent Technologies).

### 4.11 CerS1 in vitro activity assay

Ceramide synthase activity was measured in mitochondrial and microsomal fractions (20 mg) as previously described (Spassieva et al., 2006) using a reaction mix (final volume is 100 mL) containing 15 mM of C17‐sphingosine or C17‐dihydrosphingosine and 50 mM C18:0 fatty acid Co‐A in 25 mM potassium phosphate buffer (pH 7.4), as described.

### 4.12 Vertebrate animals

Vertebrate animal studies including immunohistochemistry using brain tissues were performed using protocols approved by the IACUC at the Medical University of South Carolina. The details of animal protocols and behavioral studies can be found in Supplementary Materials.

*Human specimens* Stein's VA Bedford Healthcare System Laboratory provided frozen brain tissue samples and unstained paraffin sections.

### 4.13 Statistical analyses

All data are presented as means ± SD (Standard Deviation) of at least three independent studies (*n* ≥ 3). Group comparisons were performed using Graph Pad Prism with two‐tailed unpaired *t* tests (and nonparametric tests) or a one‐way ANOVA (nonparametric or mixed). Due to relatively smaller sample sizes in animal studies, the Kruskal–Wallis test compared continuous outcomes among the three groups. Because a significant result was discovered, all possible pairwise comparisons were performed using the Wilcoxon rank‐sum test. *p* < 0.05 (*) was considered significant. For clinical analyses, the significance was calculated by the Log‐rank test. Pearson correlation coefficients were calculated using GraphPad Prism Software, 8.0.1, for correlation analysis.

## AUTHOR CONTRIBUTIONS

Conception and design: NO, OA, and BO. Methodology development: NO, OA, MFK, FCA, AA, ZMS, and BO. Data acquisition: NO, OA, MFK, and FCA. Data analysis and interpretation: NO, OA, CW, MFK, AA, and BO, Manuscript writing, review, and revision: NO and BO Administrative, technical, or material support: NO, AA, MS, ZS, and BO. Study supervision: NO, OA, and BO.

## FUNDING INFORMATION

This work was supported by funding from the National Institutes of Health (AG069769, CA214461, DE016572, and P01 CA203628 to BO) and SC SmartState Endowment in Lipidomics and Drug Discovery. MUSC SCORE CEC Scholarship for OA. The core facilities utilized are supported by NIH (C06 RR015455), Hollings Cancer Center Support Grant (P30 CA138313), or Center of Biomedical Research Excellence (Cobre) in Lipidomics and Pathobiology (P30 GM103339). The Zeiss 880 was funded by a Shared Instrumentation Grant (S10 OD018113).

## CONFLICT OF INTEREST STATEMENT

The authors declare no competing interests.

## LEAD CONTACT

Further information and requests for resources and reagents should be directed to and fulfilled by the Lead Contact, Besim Ogretmen (ogretmen@musc.edu).

## MATERIALS AVAILABILITY

All unique/stable reagents generated in this study are available from the Lead Contact with a completed Materials Transfer Agreement. The data supporting this study's findings are available upon reasonable request from the corresponding author [B.O.].

## Supporting information


Data S1.

Video S1.

## Data Availability

Data are available on request from the authors. The data supporting this study's findings are available from the corresponding author upon reasonable request.
