# Supplementary material for: Alterations of lipid‐mediated mitophagy result in aging‐dependent sensorimotor defects
Source: Aging Cell. 2023 Aug 23;22(10):e13954. doi: 10.1111/acel.13954 (PMC10577547; doi:10.1111/acel.13954)
Supplement: Supplementary file 1 — Data S1. [file ACEL-22-e13954-s002.pdf]

SUPPLEMENTAL INFORMATION

Alterations of Lipid-Mediated Mitophagy Result in Aging-Dependent Sensorimotor Defects

Natalia Oleinik[1,2#*], Onder Albayram[3,4#], Mohamed Faisal Kassir[1,2], F. Cansu Atilgan[1,2], Chase Walton[1,2], Eda Karakaya[3], John Kurtz[3], Alexander Alekseyenko[2,5], Habeeb Alsudani[6], Megan Sheridan[1,2], Zdzislaw M. Szulc[1,2], and Besim Ogretmen[1,2,*]

List of Supplementary Materials:

Supplemental Figures S1-S10

Supplementary Materials and Methods

Supplemental Figure S1

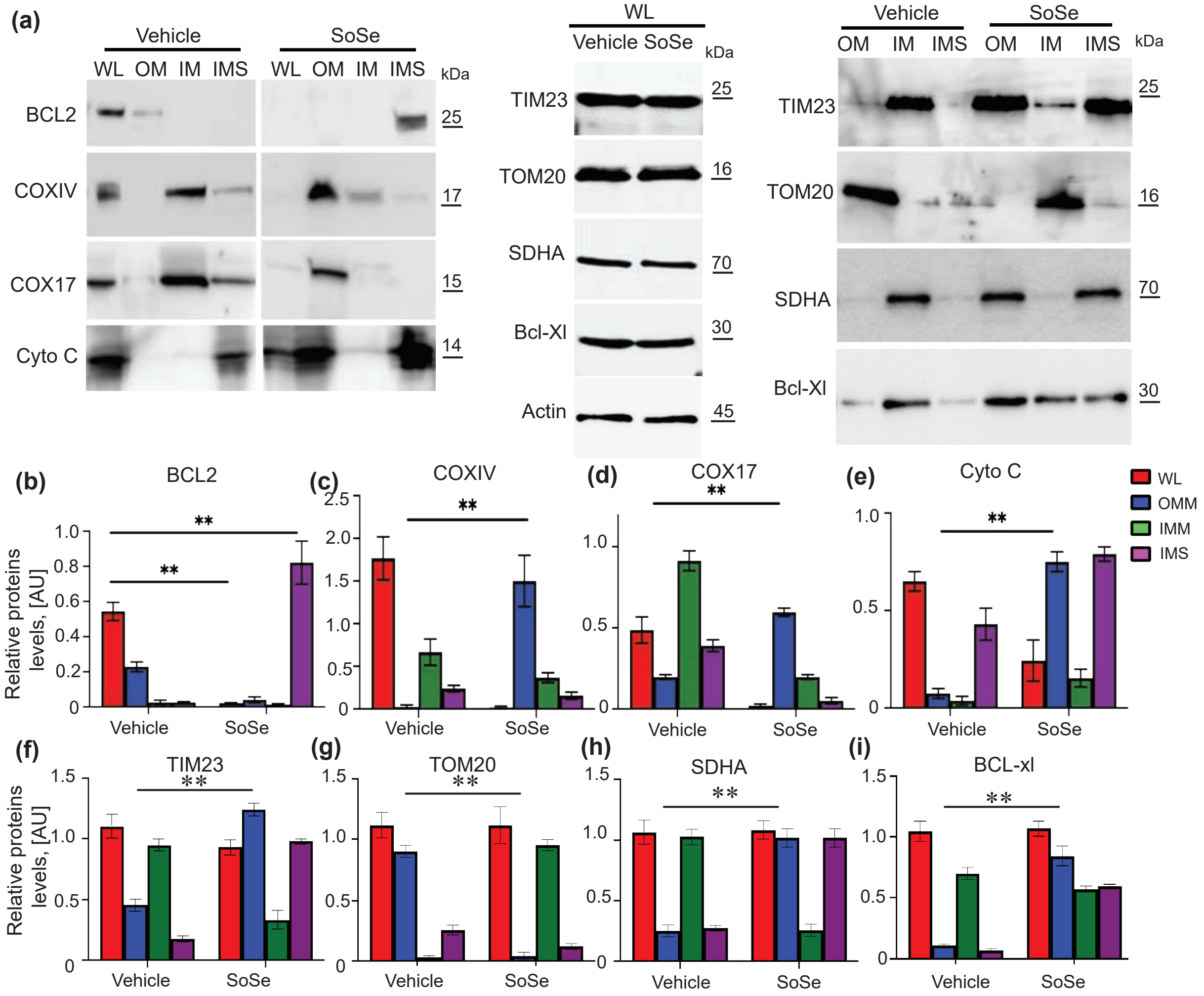

Supplemental Figure S1. SoSe treatment induces the exchange of mitochondrial proteins between OMM and IMM. a, Western blot analysis of mitochondrial proteins' distribution between OMM (outer mitochondrial membrane), IMS (intermembrane space), and IMM (inner mitochondrial membrane) in UM-SCC-1A cells treated with vehicle (left) and SoSe (right). **b-i**, Quantification of **a** was done using Fiji software. Data are means SD (n=3, *p <0.05, **p<0.01, ***p<0.001). The level of correspondent proteins in a whole lysate (WL) was used for normalization.

Supplemental Figure S2

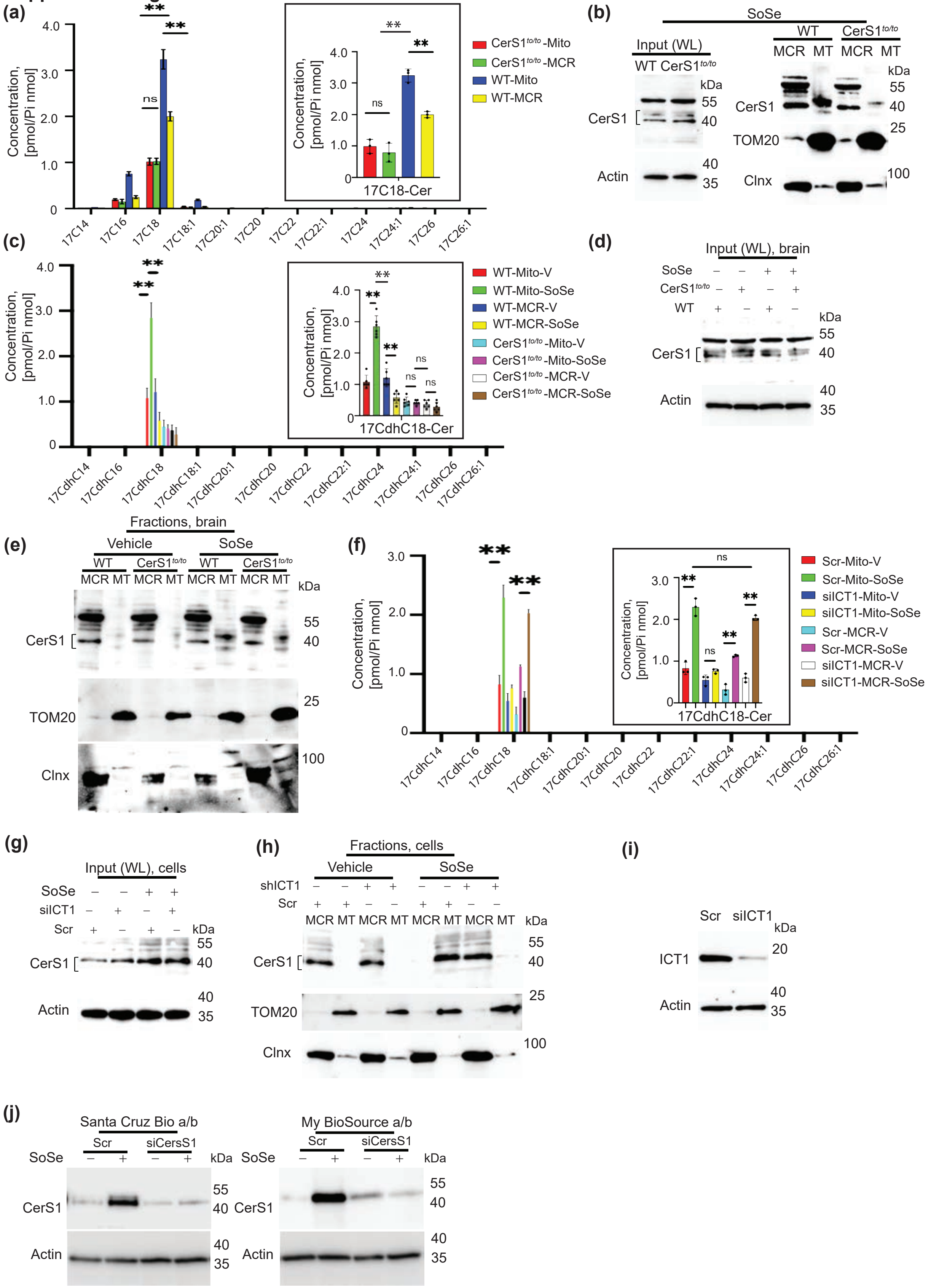

Supplemental Figure S2. CerS1 in vitro activity assay. a, Mass-spectrometry analysis of 17C18-Cer levels (left) and 17C-ceramides species synthesized *in vitro* by the mitochondrial and microsomal fractions of the brain tissue freshly isolated from CerS1$^{to/to}$ and WT mice treated with 1 mg/kg of SoSe for 3 hours. Measurements of ceramides were normalized to total inorganic phosphate in the probe. **b**, Western blot of CerS1 levels in the whole lysate (WL, input) and in microsomal and mitochondrial fractions of the brain tissues isolated from animals from **a**. Actin was used as a loading control. TOM20 and Calnexin as loading controls for mitochondrial and microsomal fractions, respectively. **c**, Mass-spectrometry analysis of 17CdhC18-Cer levels (left) and 17Cdh-ceramides species synthesized *in vitro* by the mitochondrial and microsomal fractions of the brain tissue freshly isolated from CerS1$^{to/to}$ and WT mice treated with vehicle or 1 mg/kg of SoSe for 3 hours. Measurements of ceramides were normalized to total inorganic phosphate in the probe. **d**, Western blot of CerS1 levels in the whole lysate (WL, input). Actin was used as a loading control. **e**, Levels of CerS1 in microsomal and mitochondrial fractions of the brain tissues isolated from animals from **c.** TOM20 and Calnexin as loading controls for mitochondrial and microsomal fractions, respectively. **f**, Mass-spectrometry analysis of 17CdhC18-Cer levels (left) and 17Cdh-ceramides species (right) synthesized *in vitro* by the mitochondrial and microsomal fractions of Scr control and shICT1 1A cells and treated with vehicle or SoSe (10 μM for 3 hours). Measurements of ceramides were normalized to total inorganic phosphate in the probe. **g**, Western blot of CerS1 levels in the whole lysate (WL, input). Actin was used as a loading control. **h**, CerS1 levels in microsomal and mitochondrial fractions of the 1A cells from **g.** TOM20 and Calnexin as loading controls for mitochondrial and microsomal fractions, respectively. **i**, Levels of ICT1 in Scr control and shICT1 cells. Actin was used as a loading control. **j**, Comparison of two CerS1 antibodies manufactured by Santa Cruz (# sc-65096) and MyBioSource (# MBS7104965) using

WB analysis of total lysates obtained from Scr and CerS1 siRNA transfected 1A cells and treated with vehicle or SoSe.

Supplemental Figure S3

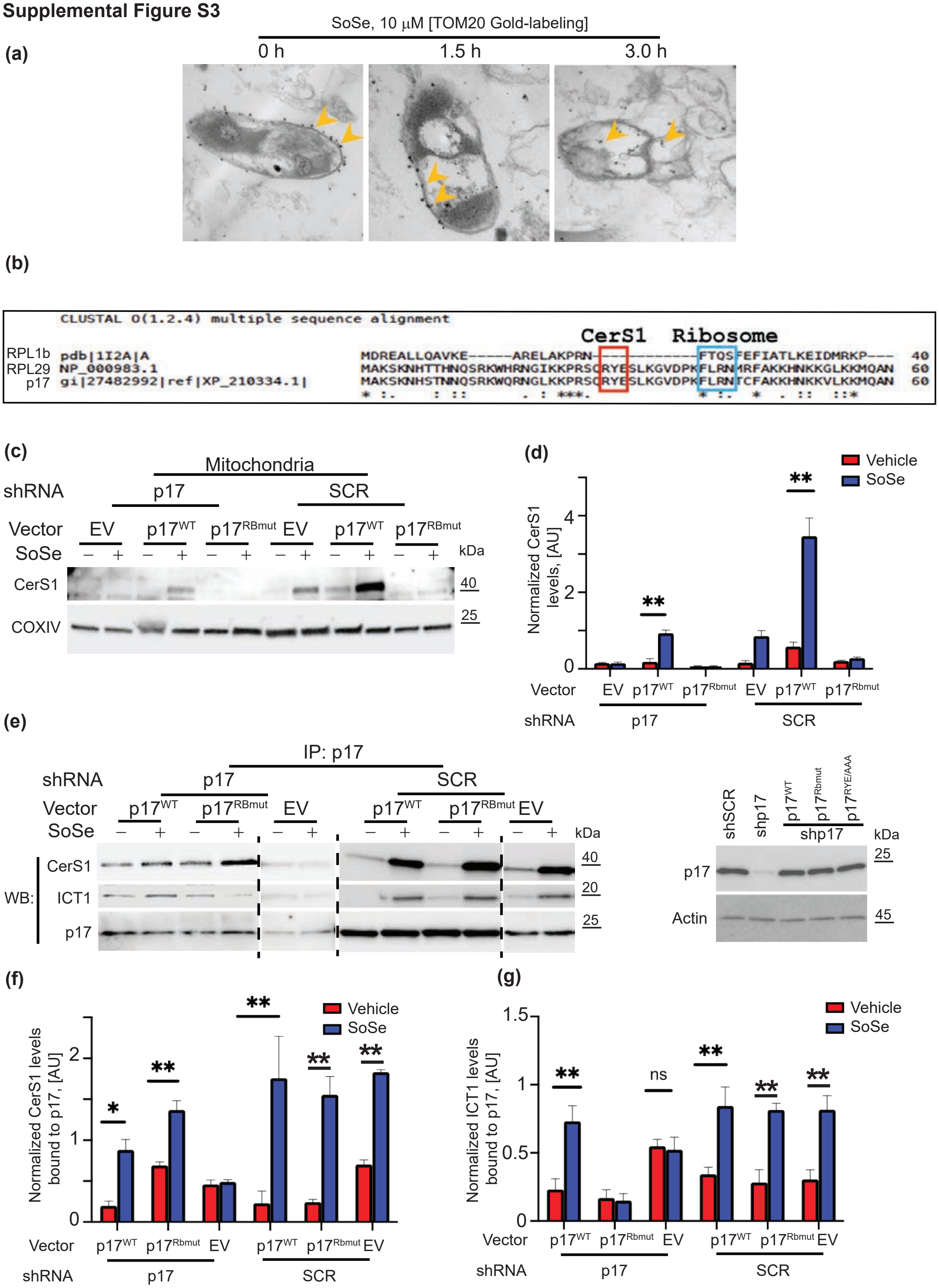

Supplemental Figure S3. SoSe-induced remodeling of mitochondrial membranes results in the misplacement of the mitochondrial membrane proteins. a, TEM images of mitochondria isolated from the UM-SCC-22A cells treated with 10 μM of SoSe and goldlabeled with anti-Tom20 antibody. Yellow arrows indicate the transition of Tom20 from the OMM at the beginning of the experiment (0 h) to IMM at the 3 h of SoSe exposure. Images represent at least three independent experiments. **b**, Identification of p17 ribosomal binding site. Multiple sequence alignment of ribosomal proteins from bacteria (L1 ribosomal protein [Methanococcus Jannaschii]; human (NP_000983.1 - 60S ribosomal protein L29 [Homo sapiens]) and p17/PERMIT. The red box indicates sequences homologs to the p17 CerS1 binding site; the blue box marks sequences responsible for ribosome binding. To inhibit p17 interaction with ribosomes, the amino acids highlighted by the blue box (Phe 39, Leu 40, Arg 41, and Asn 42) have been replaced with Ala to generate a p17 mutant lacking ribosomal binding (p17Rb). **c**, CerS1 levels in mitochondria isolated from shp17 and shScr UM-SCC-1A cells transiently expressing EV, p17WT, and p17Rb mutant and treated with SoSe (10 μM, 3 h). COXIV, a mitochondrial marker, has been used as a loading control. Images represent at least three independent experiments. **d**, Quantification of c. Data are means ± SD (n=3 independent experiments, $^{**}p < 0.01$). **e**, left panel, Co-IP analysis of p17/CerS1 and p17/ICT1 interactions in UM-SCC-1A cells from **c**. **e**, right panel, Expression levels of p17 protein in Scr control cells and shp17 stable knocked out transiently transfected with empty vector (EV), p17WT, p17 mutant lacking ribosomal recognition sequence (p17Rbmut) and p17 mutant unable to interact with CerS1 (p17RYE/AAA). **f, g**, Quantification of **e**. Data are means ± SD (n=3 independent experiments, $^{*}p < 0.05$, $^{**}p < 0.01$)

Supplemental Figure S4

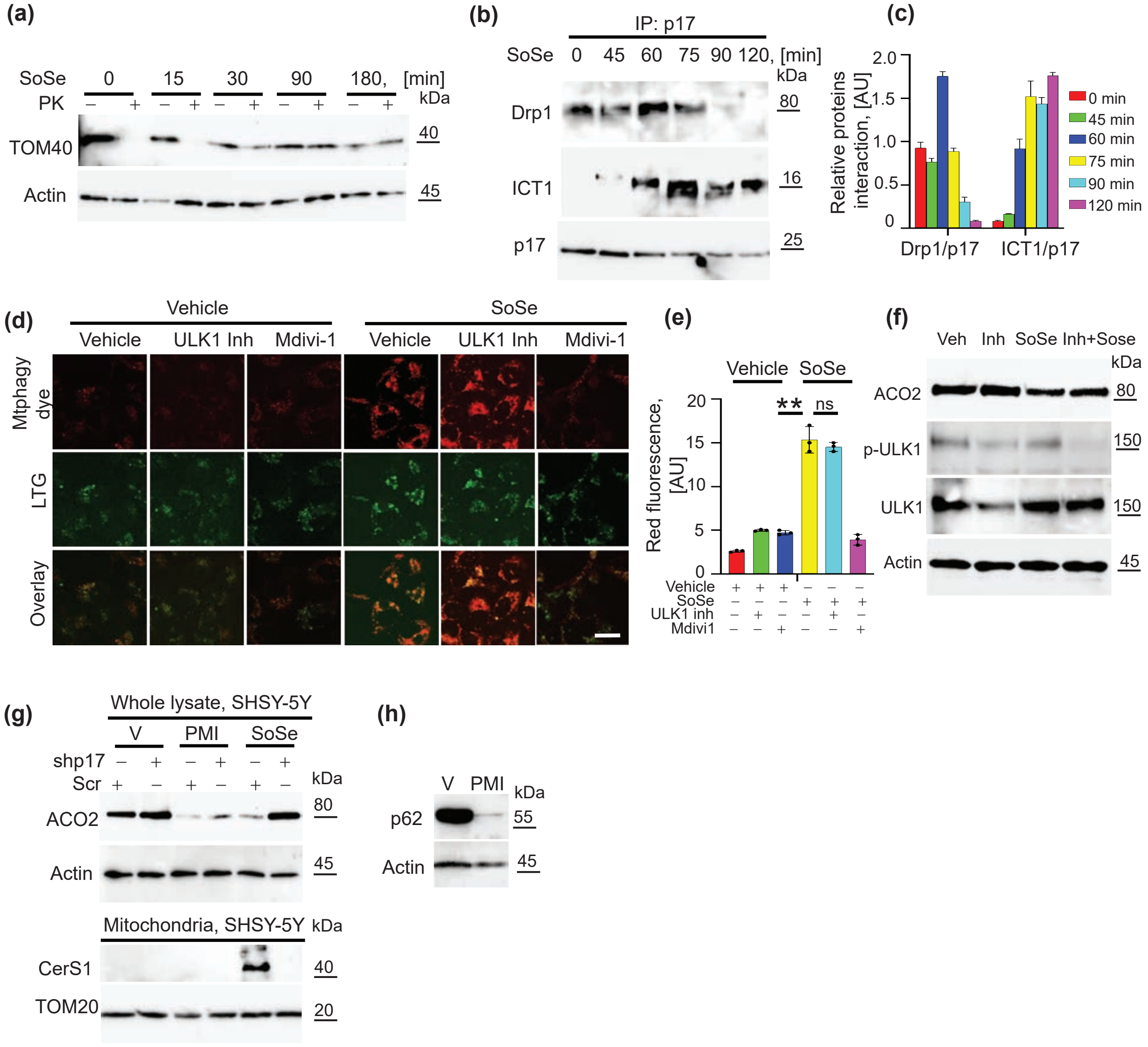

Supplemental Figure S4. a, Topological analysis by Proteinase K digestion of TOM40 in 1A cells treated with SoSe for the indicated periods (top). Actin levels in the whole lysate were used as a normalization control. **b**, co-IP analysis of time-dependent interactions between Drp1-p17/PERMIT and p17/PERMIT-ICT1. **c**, Quantification of **b**. **d**, Live cell confocal microphotographs of 1A cells labeled with Mtphagy dye (red) and Lysotracker green (LTG, green) and treated for 3 hours with 5 mM of ULK1 inhibitor (SBI-0206965) or 1 mM Mdivi1 (Drp1 inhibitor) alone or in combination with 10 mM of SoSe. **e**, Quantification of **d** using Fiji software. Data are means SD (n=3, *p <0.05, **p<0.01, ***p<0.001). **f**, ACO2, p-ULK1, and ULK-1 levels in cells from **a**. Actin was used as a loading control. **g**, Assessment of p17/PERMIT involvement in PARKIN independent mitophagic pathway. Whole lysate ACO2 levels in Scr control and sh-p17/PRRMIT SHSY-5Y cells and treated with PMI (10 mM for 24 h) and SoSe (10 mM, 3 h) (top). Bottom, mitochondrial levels of CerS1 in cells from the top panel. Actin and TOM20 were used as loading controls correspondently. **h**, Levels of p62 in SHSY-5Y cells treated with vehicle and p62-mediated mitophagy inductor (PMI, 10 mM 24 h).

Supplemental Figure S5

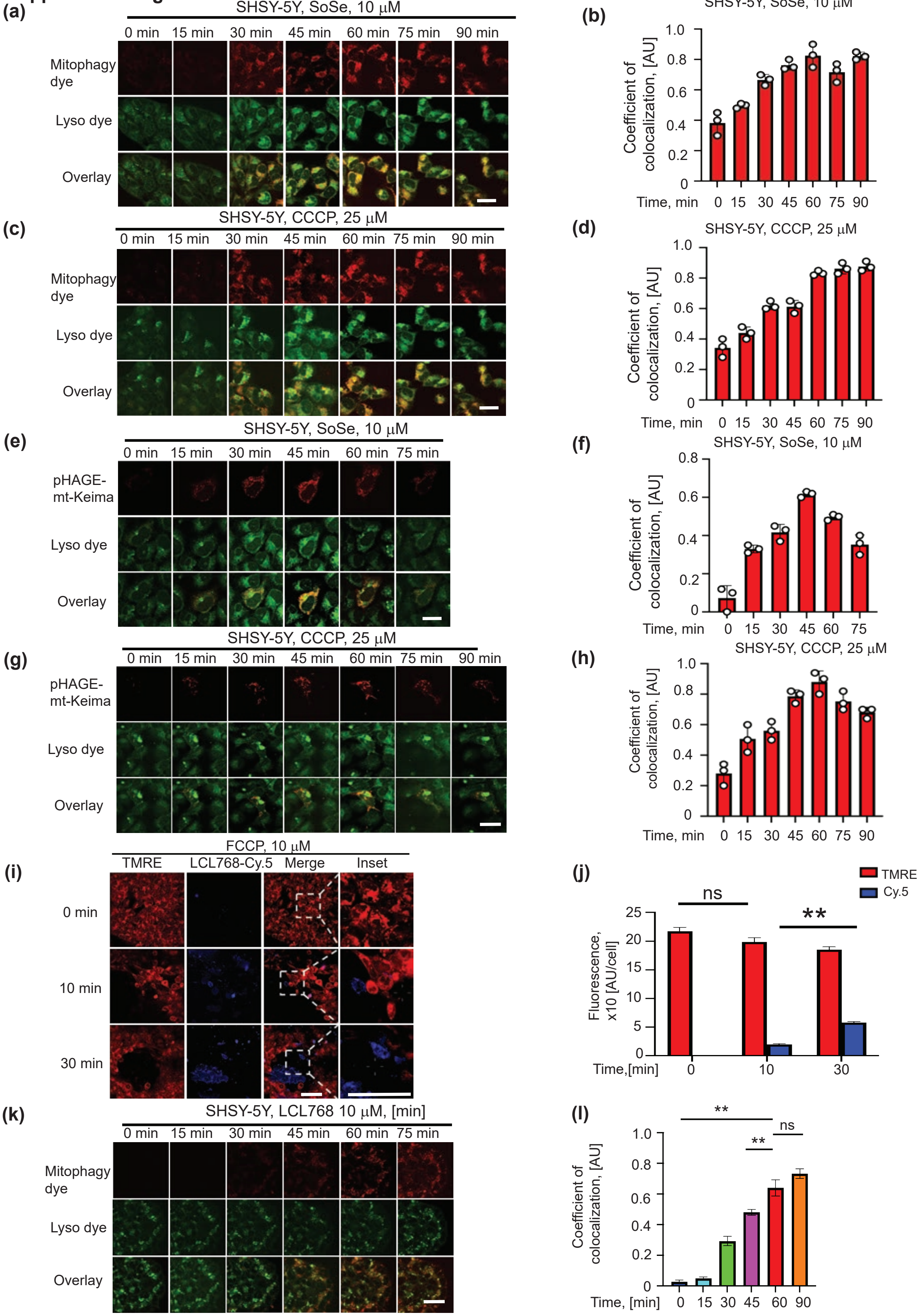

Supplemental Figure S5. Assessment of ceramide-dependent mitophagy in SHSY-5Y neurons treated with SoSe. a, Mitophagy assessment in SHSY-5Y differentiated neurons treated with SoSe (10 mM) for different times by live cell imaging using Mtphagy dye (red) and Lyso dye (green). Colocalization is shown in yellow. **b,** Quantification of the left panel by Image J. **c**, Mitophagy assessment in SHSY-5Y differentiated neurons treated with CCCP (25 mM) for indicated times by live cell imaging using Mtphagy dye (red) and Lyso dye (green). Colocalization is shown in yellow. **d**, quantification of **c** by Image J. **e,** Mitophagy assessment in SHSY-5Y differentiated neurons transiently transfected with pHAGE-mt-mKeima and treated with SoSe (10 mM) for different times by live cell imaging. Counterstain was done with Lyso dye (green). Colocalization is shown in yellow. **f** quantification of **e** by Image J. **g,** Mitophagy assessment in SHSY-5Y differentiated neurons transiently transfected with pHAGE-mt-mKeima and treated with CCCP (25 mM) for different times by live cell imaging. Counterstain was done with Lyso dye (green). Colocalization is shown in yellow. **h**, quantification of **g** by Image J. **i,** Confocal microphotographs of live SHSY-5Y cells pretreated with FCCP (10 mM for 0 min, top panel; 10 min (middle) and 30 min, bottom) and labeled with TMRE (red) and LCL768-Cy.5 (blue). Yellow dash rectangles indicate cells with mitochondria labeled with LCL768-Cy.5. **j**, Quantification of **i**. **k,** Mitophagy assessment in SHSY-5Y differentiated neurons treated with LCL768 (10 mM) for different times by live cell imaging using Mtphagy dye (red) and Lyso dye (green). Colocalization is shown in yellow. **i**, Quantification of the **k** by Image J).

Supplemental Figure S6

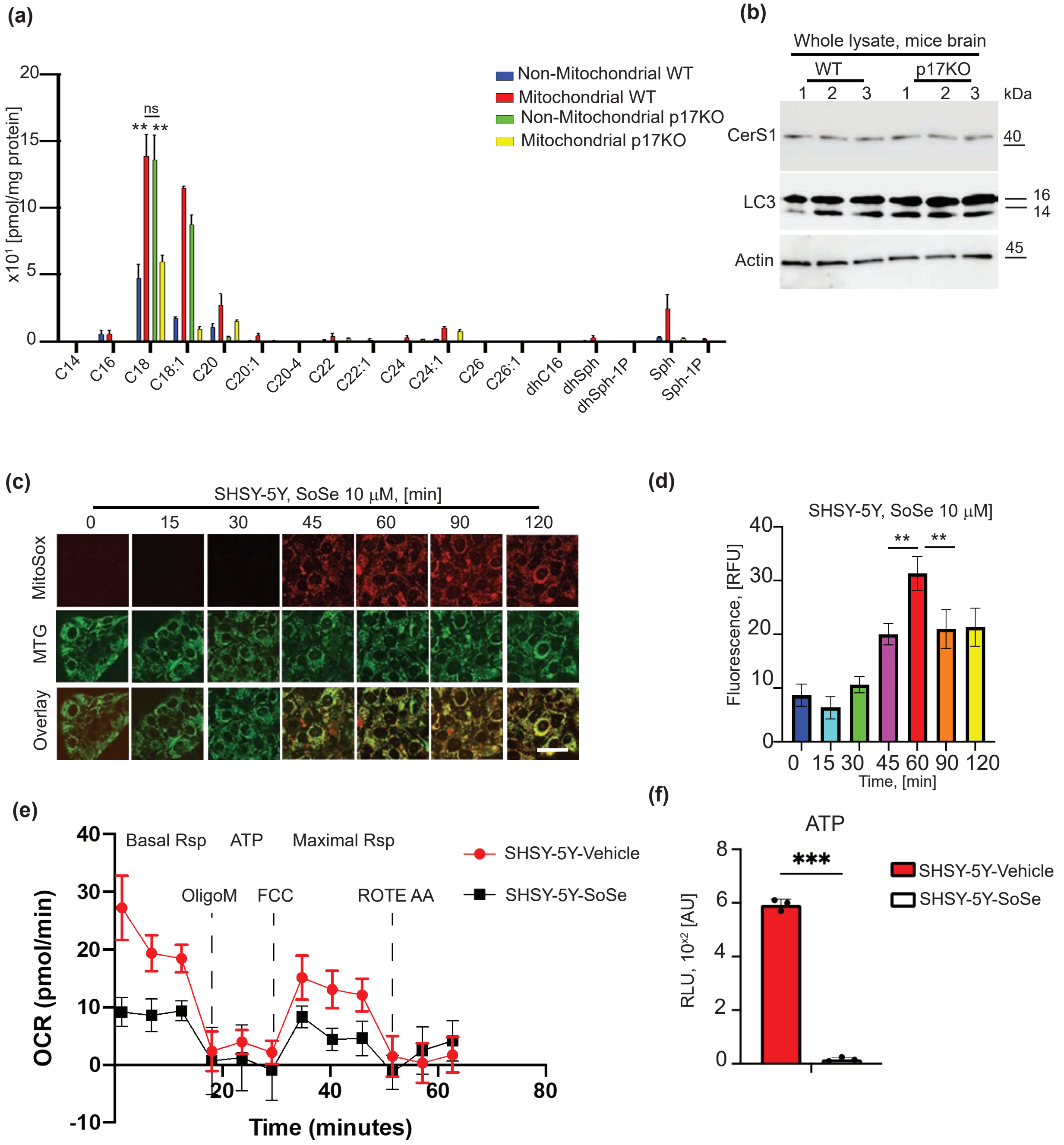

Supplemental Figure S6. LCL768 induces mitophagy in SHSY-5Y neurons. a, Lipidomics profile in mitochondrial and non-mitochondrial fractions isolated from brain tissues of WT and p17KO animals. Data are means SD (n=3, *p <0.05, **p<0.01, ***p<0.001). **b,** Levels of CerS1 and LC3 in the whole lysate of brain tissues isolated from WT and p17KO mice. **c**, Confocal images of SHSY-5Y cells treated with SoSe (10 mM, indicated periods) and labeled with MitoSox (red) and Mitochondrial Tracker Green (MTG). Yellow indicates mitochondria generating reactive oxygen species. **d**, Quantification of **c**. Data are means SD (n=3,*p <0.05, **p<0.01, ***p<0.001). **e**, Oxygen consumption rate (OCR) measured by Seahorse in SHSY-5Y cells treated with vehicle or SoSe (10 mM, 3 h). To assess key parameters of mitochondrial respiration, cells were injected with oligomycin (1 μM), FCCP (0.5 μM), and antimycin A (0.5 μM)/rotenone (0.5 μM). **f**, ATP levels in the cells from **e** were measured by CellTiter Glo Luminescent Cell Viability Assay. Data were normalized by Trypan blue cells counting and are means SD (n=3, *p <0.05, **p<0.01, ***p<0.001).

Supplemental Figure S7

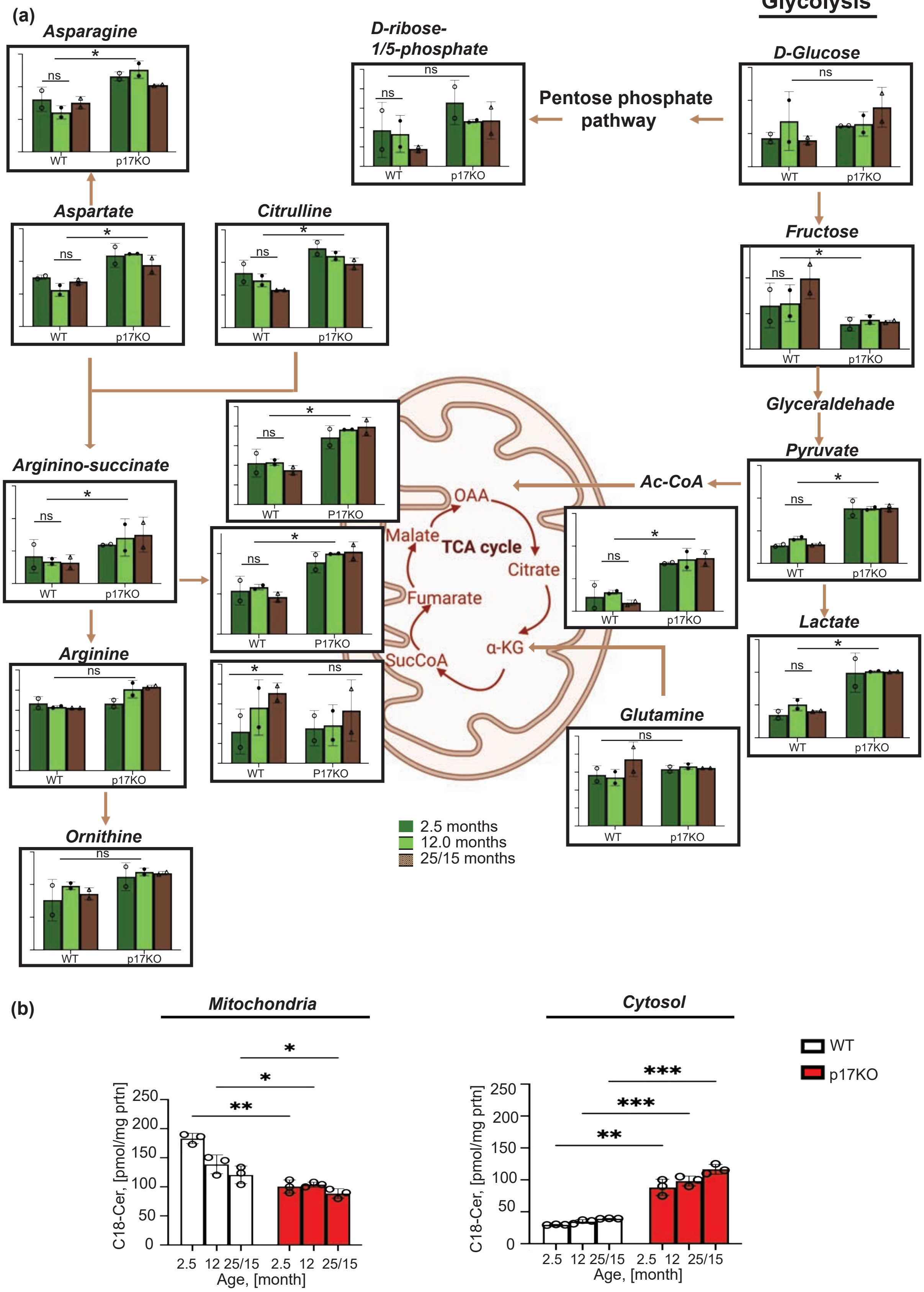

Supplemental Figure S7. Analysis of the metabolic profile of brain tissues derived from 2.5, 12, and 15/25 months old p17KO and WT mice. a, Tissue extracts were subjected to methanol extraction of water-soluble metabolites and sent for metabolomics analysis. The data were analyzed by using MetaboAnalyst 5.0 software. Data are means SD (n=5 brain tissue samples *p <0.05, **p<0.01, ***p<0.001). **b**, C18-Ceramide levels measured by mass spectrometry in mitochondria (left) and cytosol (right) of `WT and age-matched p17KO counterparts. Data are means SD (n=3 per age group *p <0.05, **p<0.01, ***p<0.001).

Supplemental Figure S8

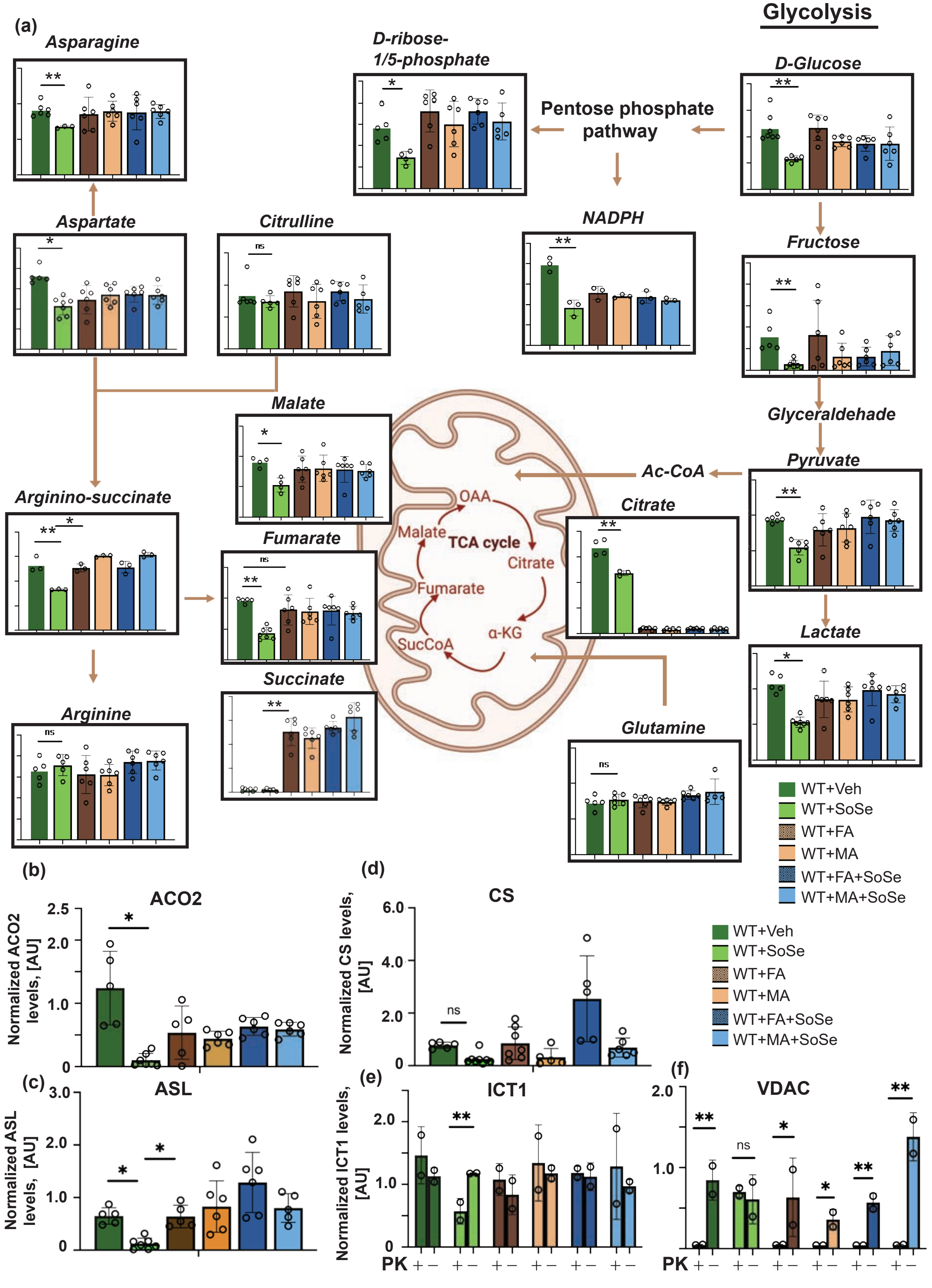

Supplemental Figure S8. Pretreatment with Fumaric and Maleic acids reverses SoSe mitophagic response in the WT animals' brains. a, Metabolomic analysis of cerebellum extracted from 1. WT mice treated with vehicle, 2. SoSe (10 μM, three hours), 3. Fumaric acid (FA, 10 mg/kg, four hours), 4. Maleic acid (MA, 10 mg/kg, four hours), 5. Fumaric acid (10 mg/kg, one hour) followed by SoSe (10 μM, three hours), 6. Maleic acid (10 mg/kg, one hour) followed by SoSe (10 μM, three hours). 8 biological replicates were subjected to methanol extraction and sent for metabolomics analysis to the Northwestern University of Chicago Feinberg School Metabolomics Core Facility. The data were analyzed by using MetaboAnalyst 5.0 software. Western blot analysis quantification of ACO2 (**b**), Argininosuccinate lyase (ASL) (**c**), and citrate synthase (CS) (**d**) levels in brain lysates of mice from **a**. Data are means ± SD (n=3 independent experiments, nsp>0.5; *p <0.05; **p<0.01; ***p<0.001). Topological analysis of the ICT1 (**e**) and VDAC (**f**) proteins by digestion with PK in mitochondria isolated from brain tissues of animals from **a**. Data are means ± SD (n=3 independent experiments, *p <0.05, **p <0.01).

Supplemental Figure S9

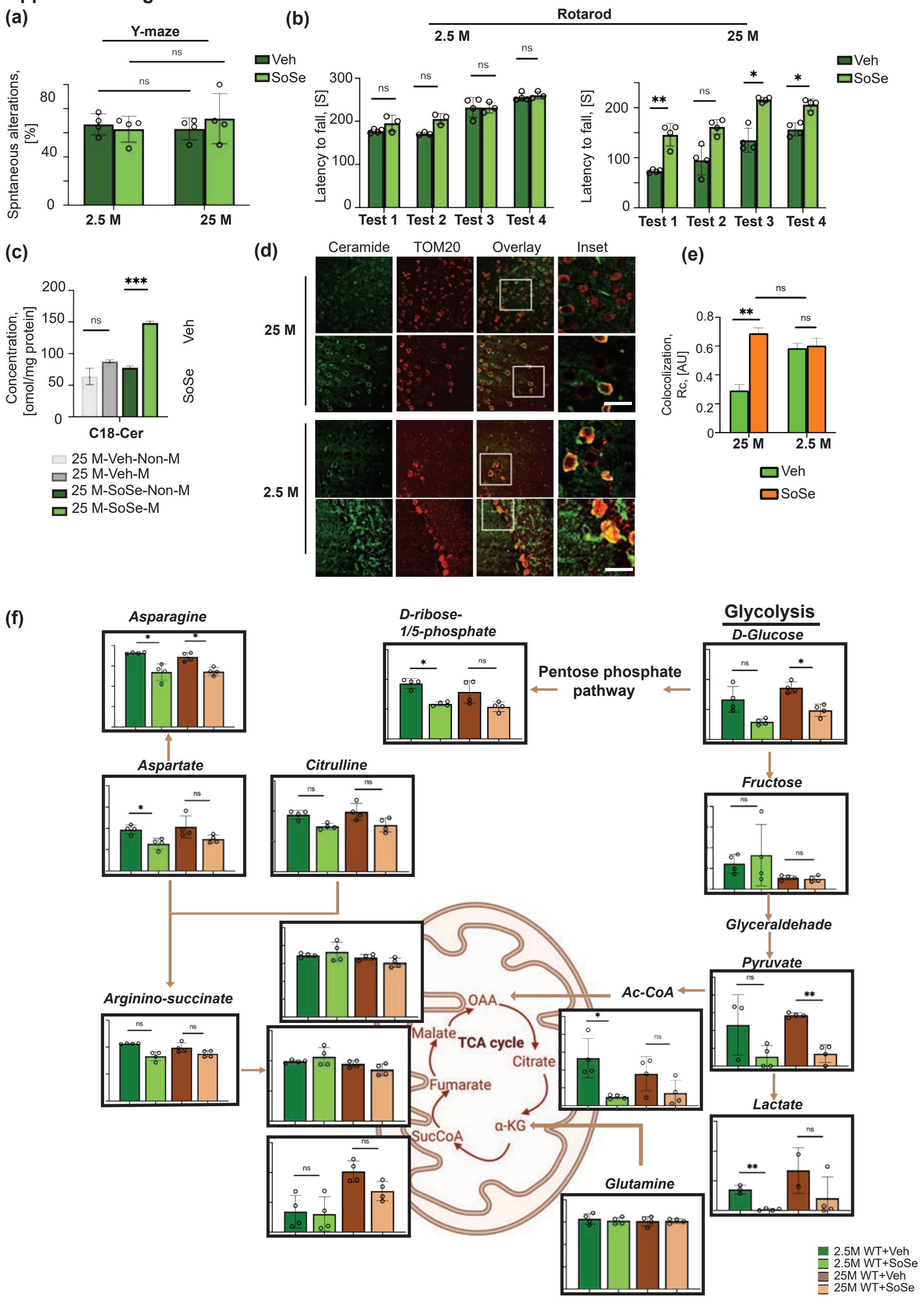

Supplemental Figure S9. SoSe treatment improves motoneuron functions in 25 months old animals by activating ceramide-dependent mitophagy. a, Evaluation of vehicle and SoSe treated 2.5- and 25-month-old mice' learning and memory by Y-Maze test. The Y-maze test was performed according to a method described elsewhere. As described below, we assessed the spontaneous alternation of entries into the three arms and working memory. Entry into an arm occurred when more than one-third of the mouse's body had entered the arm. All tests were recorded with a video camera and analyzed by an observer using Tana Move. Spontaneous alternation: All three arms were open. Mice were placed in one of the arms and allowed to explore freely for 6 min. The number and sequence of arm entries were recorded. The percentage of alternations was calculated as follows: % alternations = [number of alternations] / [total possible alternations] × 100. Working memory: One of the arms was determined as the start arm, and one was closed. Mice were placed into the start arm and allowed to explore the maze for 15 min (training trial). After a 1-h intertrial interval, all arms were opened. Mice were returned to the start arm and allowed to explore freely for 5 min (test trial). In the test trial, the number of entries in each arm, the time spent, and the first choice of entry were observed from video recordings by an observer using Tana Move. P values are indicated (nsp=0.05, (n = 4 mice per group). **b**, Evaluation of 2.5 and 25 months old C57Bl mice' motor-neuron functions by accelerated rotarod test. Latency to fall (the time mouse spent on the rotating rod with increasing velocity) of 2.5- and 25-months old WT animals treated with Vehicle or SoSe IP injections for 28 days during accelerated rotarod task for three consecutive days. The performance of 25-month-old mice treated with SoSe was significantly improved compared to vehicle-treated control animals during all three days. Repeated-measures two-way ANOVA was conducted to examine the main effect of treatment on each day (p values indicated). *p < 0.05, **p < 0.01 by t-test with Bonferroni correction. †p < 0.05

by Mann-Whitney test with Bonferroni correction (number of comparisons was ten for latency to fall). n = 4 mice per group. Error bars represent SEM. **c**, Levels of C18-and C18:1 Ceramides measured in mitochondrial (M) and non-mitochondrial (NM) fractions isolated from animals' prefrontal cortex from A measured by lipid profiling. **d**, Confocal microphotographs of prefrontal cortex isolated from animals from **a** and stained with Ceramide (green) and TOM20 (red) antibodies. The yellow signal indicates colocalization. **e**, Quantification of F. Rc, colocalization coefficient was determined using Fiji J software. Images represent at least three independent experiments. Data are means ± SD (n=3 independent experiments, **p <0.01). **f**, Metabolomic analysis of prefrontal cortex tissues extracted from 2.5- and 25-month-old WT mice treated with vehicle or SoSe (IP, 28 days). Three biological replicates of each group were subjected to methanol extraction and sent for metabolomics analysis to Northwestern University of Chicago Feinberg School Metabolomics Core Facility. The data were analyzed by using MetaboAnalyst 5.0 software.

Supplemental Figure S10

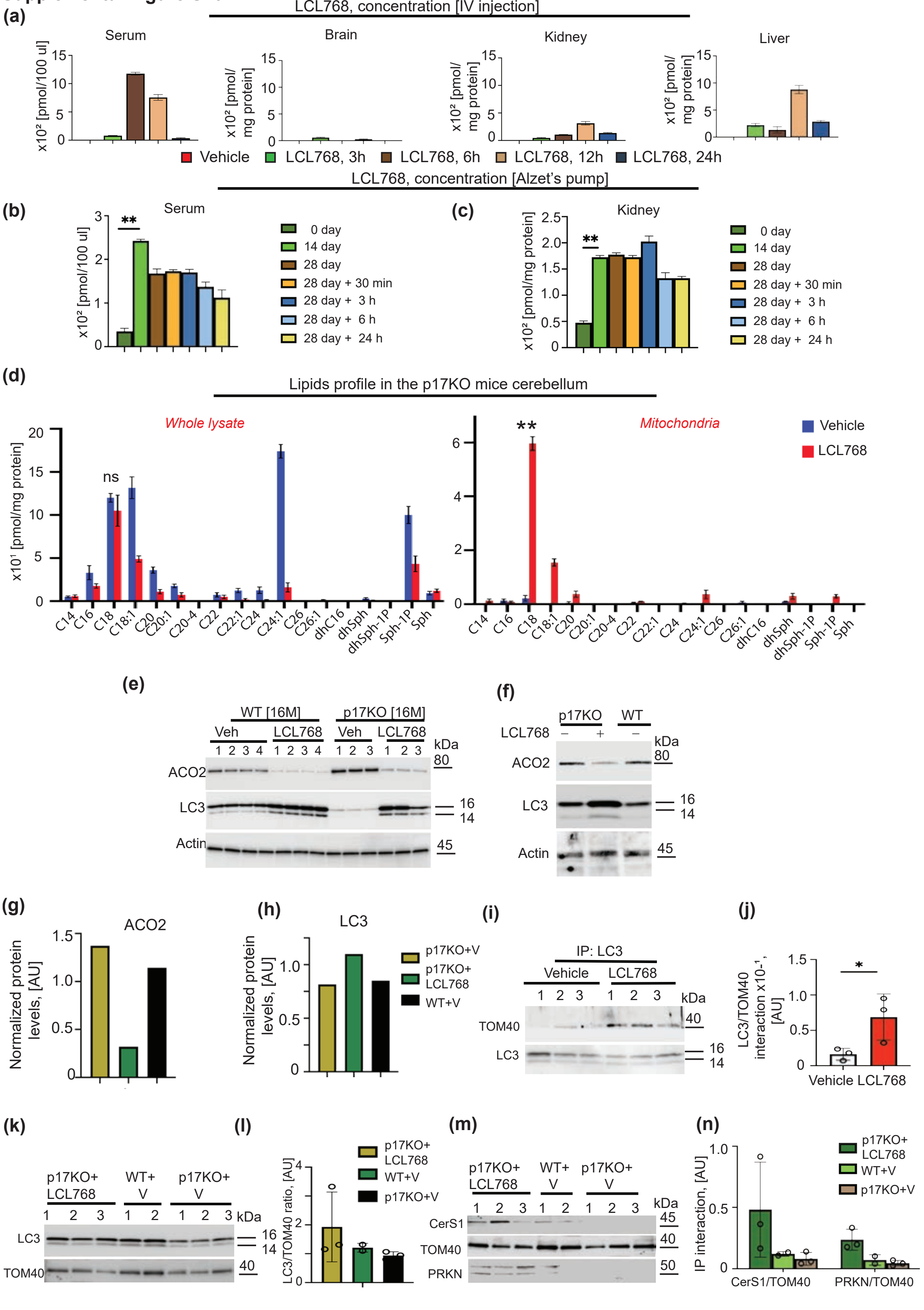

Supplemental Figure S10. Effects of LCL768 on mitophagy induction in WT and p17/PERMIT-/- mice. **a**, Concentration of LCL768 at the indicated periods in serum, brain, kidney, and liver isolated from WT animals intravenously injected with LCL768. The concentration of LCL768 during indicated periods in serum (**b**) and kidney (**c**) of p17KO animals intracranially implanted with Alzet's pumps loaded with LCLC768. Data are means ± SD (n=3, **p <0.01). **d**, Lipids profile analysis of whole lysate and mitochondrial fractions isolated from cerebellums of 16 [15+28 days] months old p17KO animals implanted either with vehicle or LCL768 for 28 days. Data are means ± SD (n=3, **p <0.01). **e**, Levels of ACO2 and LC3 in whole lysates from **b, c, f.** Levels of ACO2 and LC3 proteins in the cerebellum lysates isolated from 15 months old WT and p17KO animals implanted with Alzets pumps loaded with vehicle (-) or LCL768 (+). Actin is used as a loading control. **g, h**, Quantification of **f**. **i**, Levels of LC3 and TOM40 interaction in cerebellum isolated from 15 months old p17KO animals implanted with Alzet' pumps loaded either with vehicle (-) or LCL7868 (+) measured by co-IP. **j**, Quantification of i. Data are means ± SD (n=3, *p <0.05). Interaction between LC3 and TOM40 (**k**) and LC3, TOM40, and PARKIN (**m**) was measured by co-IP in cerebellum isolated from animals from **b**. **l**, **n** Quantification of **k** and **m** correspondently. Data are means ± SD (*p>0.05).

SUPPLEMENTARY MATERIALS and METHODS

Steady-state metabolite profiling and targeted metabolic flux analysis

The metabolic profiling was performed at the Metabolomics Core Facility of Feinberg School of Medicine at Northwestern University (Chicago, IL) by LC-MS/MS. Data were analyzed by the MetaboAnalyst V5.0 platform (https://www.metaboanalyst.ca) (Pang et al., 2021). For the flux experiments, 13C3-pyruvate (Cambridge Isotope Laboratories, #CLM-2440-1) or 13C5-glutamine (Cambridge Isotope Laboratories, #CLM-1822-H-0.1) were used. 13C3-pyruvate was prepared at a concentration of 110 mg/L in DMEM medium without pyruvate (Cytiva, #SH30022.01). 13C5-glutamine was prepared at a concentration of 584 mg/L in a DMEM medium without glutamine (Cytiva, #SH30285.01). Unlabeled cells cultured in a complete DMEM medium without a tracer were used as a control. Statistical significance was determined by a Student t-test using the Holm–Sidak method, with alpha = 5.000%. ATP was measured by CellTiter-Glo Luminescent Cell Viability Assay (Promega) according to the manufacturer's directions.

Vertebrate Animals

Vertebrate animal studies were performed using protocols approved by the IACUC at the Medical University of South Carolina as follows: **SoSe treatments (acute and chronic) in mice:** Vehicle or 1 mg/kg for 3 h of SoSe (acute) or 0.5 mg/kg for three days/week for 28 days (chronic) injected intraperitoneally (IP). **Fumarate and malate treatments in mice:** Six groups (8 animals/each) of 3-4 months old C57BL/6 males have been IP injected: Vehicle, 1mg/kg SoSe for three hours; Fumaric acid (FA), 10 mg/kg; Malic acid (MA), FA (10 mg/kg) pretreatment for one hour before three hours treatment with 1 mg/kg of SoSe (FA+SoSe) and MA (10 mg/kg)) pretreatment for 1 hour before three hours treatment with 1 mg/kg of SoSe (MA+SoSe). The total treatment was for

four hours. **Alzet pumps' intracranial implantation and LCL768 treatment in mice:** For the procedure, we used Alzet mini-osmotic pump model 2004 and brain infusion kit #3 according to the manufacturer's instructions (http//www. jove.com/vodeo/50326; DOI: doi:10.3791/50326). The pump was assembled, loaded with LCL768 (1 mg/kg; 0.25 ml/h for 28 days), and equilibrated for 48 h at 37 oC. This was followed by intracranial implantation as described by the manufacturer. **Behavioral studies in mice. Accelerated Rotarod test:** The mice were placed in the rotating cylinder twice daily for three days. Each trial lasts a maximum of 10 min, during which the rotating rod accelerates from 4 to 40 rpm over the first 5 min of the trial and then remains at maximum speed for the remaining 5 min. Animals rested for at least 10 min between trails to avoid fatigue and exhaustion. **Morris Water Maze Test**: Over five days, animals were tested for four sessions daily. The hidden platform remained at a fixed spatial location for the entire acquisition period (5 days). Mice were released facing the wall of the maze at each session of the trial. Four equally distributed points along the wall served as starting points (N, S, E, and W) and divided the area into four quadrants. During the experiment, mice were constantly monitored and traced with a computer-assisted video tracking system (Noldus Ethovision). **Elevated Plus Maze test**: Mice were placed on the center platform of the maze, facing a closed arm, and allowed to explore the apparatus for 5 minutes. Time spent in the open area, distance traveled in the open and closed parts, and the number of stretching postures were evaluated. **Bright-Light Open Field Test:** Mice were placed in the center of the open-field apparatus' brightly lit (200–300 lux) chamber (44x44x30 cm). An automatic monitoring system tracked the movements of the animals for 5 min. Horizontal motor (distance traveled) and main activity (distance traveled in central area/ total distance traveled) were evaluated. **Novelty Preference test (Y-maze):** This test included two trials. During Trial 1, one of the arms of the Y-maze was blocked, allowing for a three min

exploration of only two arms of the maze. After a 1 min delay, Trial 2 was started. During Trial 2, all three arms were available for another 3 min of exploration. Trial 2 took advantage of the innate tendency of mice to explore novel unexplored areas (e.g., the previously blocked arm). The time spent in novel new areas of each animal was measured. Mice with intact short-term memory prefer to explore a novel arm over the familiar arms, whereas mice with impaired episodic memory enter all arms randomly. **String Suspension test:** Mice were permitted to grasp the string only by their forepaws and then released. Each mouse was tested three times (each test takes 20 sec) and scored from 0 to 3 depending on the severity of deficits. **Ledge Assay:** Mice were placed on the cage's ledge and monitored. Each mouse was tested two times (each test takes 20 seconds). If a mouse walked along the ledge and back into the cage delicately, the score of 0; if it fell off the ledge or avoided walking, a score of 3 was recorded.

Immunohistochemistry

Upon animal sacrifice, the cerebellum was collected and placed for 24 hours in a 10% formaldehyde solution, followed by incubation in 70% ethanol. After treatment with 0.3 % hydrogen peroxide, slides were briefly boiled in 10 mM sodium citrate, pH 6.0, for antigen enhancement. The sections were incubated with primary antibodies overnight at 4°C. Labeled sections were visualized with a Zeiss confocal microscope or the Keyence X800.
